# Genetic and Phenotypic Associations With Sustained Antidepressant Use in Major Depressive Disorder

**DOI:** 10.1001/jamapsychiatry.2025.4372

**Published:** 2026-01-28

**Authors:** Alicia Walker, Brittany L. Mitchell, Tian Lin, Jacob J. Crouse, Clara Albiñana, Chloe X. Yap, Mary Ellen Lynall, Penelope A. Lind, Andrea Cipriani, Enda M. Byrne, Sarah E. Medland, Nicholas G. Martin, Maxime Taquet, Ian B. Hickie, Naomi R. Wray

**Affiliations:** 1 Department of Psychiatry, Warneford Hospital, University of Oxford, Oxford, United Kingdom; 2 Brain and Mental Health Program, QIMR Berghofer Medical Research Institute, Brisbane, Queensland, Australia; 3 School of Biomedical Sciences, The University of Queensland, Brisbane, Queensland, Australia; 4 School of Biomedical Sciences, Queensland University of Technology, Brisbane, Queensland, Australia; 5 Institute for Molecular Bioscience, The University of Queensland, Brisbane, Queensland, Australia; 6 Brain and Mind Centre, The University of Sydney, Sydney, New South Wales, Australia; 7 Mater Research Institute, The University of Queensland, Brisbane, Queensland, Australia; 8 Department of Psychiatry, University of Cambridge, Cambridge, United Kingdom; 9 Psychiatric Genetics, Queensland Institute of Medical Research Berghofer Medical Research Institute, Brisbane, Queensland, Australia; 10 Oxford Precision Psychiatry Lab, National Institute for Health and Care Research Oxford Health Biomedical Research Centre, Oxford, United Kingdom; 11 NIHR Oxford Health Clinical Research Facility, Oxford Health National Health Service Foundation Trust, Oxford, United Kingdom; 12 Child Health Research Centre, The University of Queensland, Brisbane, Queensland, Australia; 13 School of Psychology, The University of Queensland, Brisbane, Queensland, Australia; 14 School of Psychology and Counselling, Queensland University of Technology, Brisbane, Queensland, Australia; 15 Genetic Epidemiology, Queensland Institute of Medical Research Berghofer Medical Research Institute, Brisbane, Queensland, Australia

## Abstract

**Importance:**

Antidepressant treatment remains a trial-and-error process: one-third of people with major depressive disorder (MDD) report inefficacy of first-line medications. Predictors of prescription patterns are needed to improve prescribing precision.

**Objective:**

To investigate phenotypic and genetic heterogeneity of MDD subgroups defined by antidepressant prescription patterns.

**Design, Setting, and Participants:**

This was a retrospective cohort study of Australian Genetics of Depression Study (2017-2018) adult participants (aged ≥18 years) with lifetime MDD who filled 1 or more prescriptions of the 10 most commonly used antidepressants across 4.5 years (2013-2017). Data were analyzed from August 2024 to October 2025.

**Exposures:**

Treatment complexity was assessed as number of different antidepressant classes in prescriptions filled in 4.5 years. Sustained-use 360 groups were defined as 360 or more cumulative days (in 4.5 years) of a single antidepressant. Participants with genome-wide genotypes were contrasted across mutually exclusive sustained-use 360 groups.

**Main Outcomes and Measures:**

Associations of 44 self-reported phenotypes and polygenic scores (PGSs) for 15 traits with sustained-use 360 subgroups. Genome-wide association studies (GWASs) were conducted for selective serotonin reuptake inhibitor (SSRI) or SSRI/serotonin-norepinephrine reuptake inhibitor sustained use contrasted to other participants.

**Results:**

Of 12 074 participants (9041 [75%] female with a mean [SD] age of 41.8 [14.6] years; 3022 [25%] male with a mean [SD] age of 47.7 [14.6] years) with 1 or more prescriptions and lifetime MDD, 8898 had genotyping data. High treatment complexity was significantly associated with 37 of 44 self-reported phenotypes (eg, higher rates of smoking, recurrent MDD, suicidal ideation, chronic pain, and circadian and atypical depression subtypes) and higher PGSs for psychiatric traits (MDD PGS: β, 0.04; 95% CI, 0.03-0.06; *P* = 1.2 × 10^−8^; ADHD PGS: β, 0.03; 95% CI, 0.02-0.05; *P* = 2.1 × 10^−5 ^; bipolar disorder PGS: β, 0.03; 95% CI, 0.01-0.04. *P* = 1.2 × 10^−4 ^; neuroticism PGS: β, 0.02; 95% CI, 0.01-0.04; *P* = 1.3 × 10^−3^). A total of 5453 (61%) met criteria for an exclusive antidepressant sustained-use 360 group. These groups had distinct phenotypic profiles, including associations with body mass index, suicidal ideation, and co-occurring conditions. GWASs identified novel loci, including an immune-related gene *SLAMF3*/*LY9*, for which single-nucleotide variant rs4656934 was associated with reduced odds of sustained SSRI use (G allele; odds ratio, 0.81; 95% CI, 0.75-0.87; *P* = 3.5 × 10^−8^).

**Conclusions and Relevance:**

This study found that phenotypic factors were associated with sustained antidepressant use and treatment complexity. PGSs for traits studied were associated with treatment complexity but showed little association with sustained-use 360 groups. These findings support further research to guide treatment selection and to identify patients at risk of difficult-to-treat depression, informing precision psychiatry and early intervention in MDD.

## Key Points

**Question:**

Can real-world antidepressant prescription patterns identify biologically meaningful subtypes of major depressive disorder (MDD) and inform precision psychiatry approaches?

**Findings:**

In this cohort study of 12 074 participants, MDD subgroups defined by sustained single-drug antidepressant use (≥360 days) had distinct phenotypic profiles; polygenic scores for psychiatric disorders differentiated individuals with greater class diversity but not single-drug sustained-use 360 groups. One genome-wide significant immune-related variant in *SLAMF3*/*LY9* was associated with sustained selective serotonin reuptake inhibitor use.

**Meaning:**

The findings suggest that real-world antidepressant prescription patterns have the potential to identify phenotypically distinct MDD subtypes that may guide personalized treatment selection, and polygenic scores may help identify which patients are at risk of difficult-to-treat depression.

## Teaser

This cohort study evaluates phenotypic and genetic heterogeneity in major depressive disorder.

## Introduction

Major depressive disorder (MDD) affects approximately 16% of people worldwide.^1^ While antidepressants are more effective than placebo,^2^ treatment selection remains empirical (using a trial-and-error approach). Australian prescribing guidelines (Royal Australian and New Zealand College of Psychiatrists [RANZCP], 2020)^3^ recommend sequential trial of 7 first-line antidepressants, each of a different class, while earlier (2015) guidelines recommended selective serotonin reuptake inhibitors (SSRIs)/serotonin-norepinephrine reuptake inhibitors (SNRIs) as first-line medications.^4^ Despite such structured approaches, international response rates remain suboptimal.^5,6,7^ In the Australian Genetics of Depression Study (AGDS; recruited 2017-2018), one-third of users rated each common antidepressant as ineffective, with 42% self-reporting having trialed 3 or more antidepressants,^7^ highlighting the need for better predictors of treatment response.

Antidepressant outcomes likely reflect complex drug-patient interactions. Genome-wide association studies (GWASs) have estimated that 13% to 25% of variance in antidepressant response^8,9^ and 8% in treatment-resistant depression^10^ is attributable to common genetic variation (when response is assessed using clinical or self-report measures). Although few individual genome-wide significant variants have been identified for treatment-resistant depression, polygenic scores (PGSs) aggregating small genetic effects could be used in precision medicine approaches.^11^ Cross-disorder genetic effects may also inform treatment prediction; for example, in bipolar disorder (BIP), a higher schizophrenia PGS had been found to be associated with poorer lithium response.^12^

Prior antidepressant response research using clinical outcomes (eg, remission and symptom improvement) or self-report scales^8,9,13,14,15^ may miss real-world treatment response indicators. Prescription databases, in principle, provide objective, scalable assessments of medication use and proxy outcomes.^16^ Studies using prescription data typically approximate response via switching patterns or sustained use,^16,17,18,19^ though treatment-resistant depression definitions vary.^10,17^ To our knowledge, no study has compared genetic profiles across individuals with sustained use of distinct antidepressants.

This study uses AGDS data linked to 4.5 years of national prescription records to investigate phenotypic and genetic heterogeneity of MDD subgroups defined by prescription patterns. Individuals with 360 or more cumulative days in 4.5 years of single-drug antidepressant scripts were defined as having sustained-use 360. We also explored features associated with difficult-to-treat depression,^20,21^ operationalized as diversity of antidepressants and drug classes, and cumulative treatment duration, to reflect treatment complexity and intensity. This represents the first use of AGDS prescription data to assess treatment outcomes, extending beyond previous self-report-based studies.^22,23,24,25^

## Methods

### Study Population and Data Sources

The Australian Genetics of Depression Study (AGDS)^7,26^ is a national volunteer cohort study comprising individuals with self-reported depression (details in Byrne et al^7^). During initial data collection, between April 2017 and March 2018, 22 414 participants aged 18 years and older completed online questionnaires assessing psychosocial factors, lifetime MDD (via the Composite International Diagnostic Interview Short Form [CIDI-SF]), and responses to the 10 most commonly prescribed antidepressants in Australia. Of these participants, 19 291 (88.9%) met criteria for lifetime MDD, with 724 (3.2%) having missing CIDI-SF data. Participants provided saliva samples for genetic analysis using the Illumina Global Screening Array, with 14 501 individuals of genetically inferred European ancestry passing quality control.^27^ A total of 16 657 AGDS participants consented to linkage with Australian Pharmaceutical Benefits Scheme (PBS) data through Services Australia, providing prescription data from July 1, 2013, to December 31, 2017. Ethical approval for this study was obtained from the QIMR Berghofer Human Research Ethics Committee.

### Study Medications, Selection Criteria, and Cumulative Prescription Duration Estimation

We focused on the 10 most commonly prescribed antidepressants in Australia spanning 4 classes: (1) SSRIs: citalopram, escitalopram, paroxetine, sertraline, fluoxetine; (2) SNRIs: desvenlafaxine, duloxetine, venlafaxine; (3) tricyclics (TCAs): amitriptyline; and (4) tetracyclics (TeCAs): mirtazapine. Criteria for selection into this study were PBS linkage, lifetime MDD diagnosis, and 1 or more prescriptions of study medications, resulting in 12 074 participants.

Owing to the complexity of dosage reporting in PBS data, we initially assumed each prescription covered 30 days, based on typical Australian prescribing patterns. We then refined this estimate by using the lesser of 30 days or the interval until the next dispense of the same medication. This prevents overestimation of treatment duration due to early refills or overlapping prescriptions used to achieve higher doses. This approach emphasizes treatment duration rather than dosage intensity. Cumulative duration was calculated as the sum of individual prescription durations at the drug level, allowing for natural treatment gaps between prescription episodes. Prescription episodes were defined as continuous medication dispensing periods, with new episodes beginning when gaps exceeded 90 days. We assessed treatment complexity using 3 metrics: cumulative duration across all study antidepressants, antidepressant diversity (number of unique antidepressants dispensed), and class diversity (number of unique antidepressant classes dispensed).

### Defining Sustained Use

We defined sustained-use 360 as 360 or more cumulative days of a single antidepressant, based on RANZCP^3^ and National Institute for Health and Care Excellence^28^ guidelines recommending 6 or more months of maintenance therapy to prevent relapse following initial treatment. The 360-day threshold (approximately 12 months) conservatively captures both acute treatment and maintenance phases, representing a complete treatment cycle. This cumulative approach reflects real-world prescribing, including noncontinuous but recurrent use (eFigure 1 in Supplement 1), and is more stringent than common stable response cutoffs (≥180 days),^29^ while clinical trials often define acceptability by discontinuation rates after shorter periods (eg, 8 weeks).^2,30^ Sensitivity analyses used thresholds of 600 or more days (sustained-use 600) and high-continuity (≥360 days, ≤90-day gaps) to examine extended treatment and ensure findings reflect genuine population differences rather than adherence behaviors.

### Treatment Group Classification

We focused on 8898 genotyped participants with European ancestry, lifetime MDD and 1 or more antidepressant prescriptions, and assignation to mutually exclusive treatment subgroups based on prescription patterns (eFigure 1 in Supplement 1). Although AGDS participants were recruited on the basis of depression, some also self-reported a diagnosis of BIP. To avoid bias from purposeful prescribing patterns in BIP (eg, intermittent antidepressant use with mood stabilizers), these individuals were analyzed separately. We further separated this BIP subgroup into those with or without prescription records of lithium, as the former more clearly indicates a diagnosis of BIP.

This resulted in 5 groupings: (1) single-antidepressant sustained-use 360 without self-reported BIP which was further stratified by specific drug; (2) combination: sustained use of multiple antidepressants across the 4.5-year period without self-reported BIP; (3) various: other dispensing patterns without self-reported BIP; (4) BIP with sustained lithium treatment (BIP+L: dispensed >3 lithium prescriptions over the 4.5-year period of pharmaceutical data); (5) BIP without sustained lithium treatment (BIP−L: dispensed ≤3 lithium prescriptions). For analyses at the class level, the first group was collated by antidepressant class, based on sustained-use 360 of 1 or more antidepressants within the same class, while those with sustained-use 360 across multiple classes were reallocated to the various group at the class level since no class-level combination category was defined.

### Statistical Analysis

We conducted 2 sets of regression analyses: (1) treatment complexity metrics (ie, cumulative duration, class diversity, and antidepressant diversity) as outcomes regressed on 1 of 15 PGSs (eTable 1 in Supplement 2) or 59 phenotypes (44 self-reported, 15 prescription-derived), with covariates age, sex, and principal components (for PGS only) and (2) sustained-use 360 groups compared using SSRI/sertraline users as reference, with 15 PGSs and 64 phenotypes as outcomes, with covariates age and sex (self-reported phenotypes only) and principal components (PGS only). Analysis 1 was repeated sex-stratified for self-reported phenotypes and PGS traits. PGS single-nucleotide variant (SNV) weights were calculated using SBayesRC,^31^ applied to published GWAS summary statistics. Full details, and derivations of self-reported and prescription-derived phenotypes, including general adherence scores, coprescribing patterns, and antipsychotic augmentation, are detailed in the eMethods in Supplement 1 and eTable 2 in Supplement 2. Multiple testing corrections were applied separately for each treatment group and each treatment complexity metric, accounting for the number of traits tested within each analysis. Data were analyzed from August 2024 to October 2025.

## Results

### Antidepressant Landscape of the AGDS

**Figure 1.**
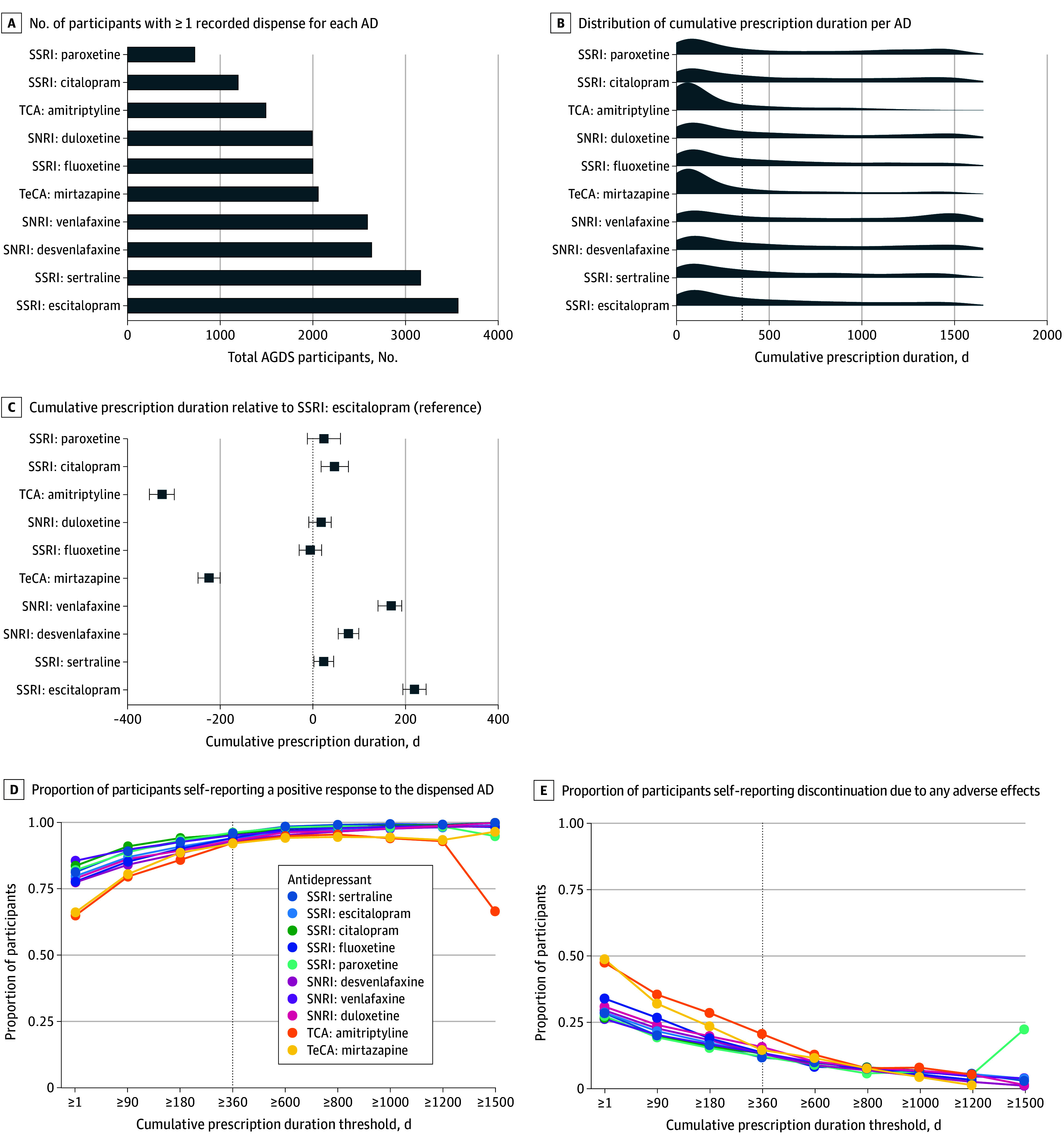
Antidepressant (AD) Use of the Australian Genetics of Depression Study (AGDS) Data are shown for 12 076 AGDS participants with lifetime depression who had 1 or more recorded dispense for one of the 10 most commonly prescribed ADs in Australia, based on linked Pharmaceutical Benefits Scheme (PBS) records from July 2013 to December 2018. A, Number of participants with 1 or more recorded dispense for each AD. B, Distribution of cumulative prescription duration per AD. The dashed line indicates sustained-use 360 (defined as ≥360 cumulative days of a single antidepressant), used as the primary threshold for sustained use. C, Cumulative prescription duration relative to the selective serotonin reuptake inhibitor (SSRI) escitalopram (reference), estimated using a linear mixed-effects model with participant as a random effect and age and sex as fixed effect covariates. D, Proportion of participants self-reporting a positive response to the dispensed AD, stratified by increasing thresholds of cumulative prescription duration (range: ≥1 to ≥1500 days). Responses of moderately well or very well were classified as responders; not at all as nonresponders. E, Proportion of participants self-reporting discontinuation due to any adverse effects, also stratified by cumulative prescription duration (range: ≥1 to ≥1500 days). Dashed lines in panels D and E indicate the sustained-use 360 threshold. SSRI indicates selective serotonin reuptake inhibitor; SNRI, serotonin-norepinephrine reuptake inhibitor; TCA, tricyclic antidepressants; TeCA, tetracyclic antidepressants.

Among the 10 most frequently dispensed antidepressants (12 074 AGDS participants), SSRIs and SNRIs were the most common, reflecting the 2015 Australian prescribing guidelines (RANZCP) contemporary to the study period.^4^ TCAs and TeCAs had shorter cumulative durations, likely reflecting their limited clinical use (Figure 1A-C; eTable 3 in Supplement 2; class level results in eFigure 2 in Supplement 1). Rates of self-reported response and discontinuation due to all causes varied by cumulative duration (Figure 1D-E).

### Phenotypic and Comedication Factors Associated With Treatment Complexity

Of 12 074 individuals, 9041 were (75%) female with a mean (SD) age of 41.8 (14.6) years, and 3022 (25%) were male with a mean (SD) age of 47.7 (14.6) years. Of 59 phenotypes (44 self-reported, 15 prescription-derived), 30 self-reported phenotypes were associated with longer cumulative duration and 37 with greater antidepressant class diversity after false discovery rate correction (eFigure 3 in Supplement 1, eTable 4 in Supplement 2). Associations formed 3 patterns: traits linked only to cumulative duration (eg, type 2 diabetes), only to increased class diversity (eg, smoking, alcohol consumption, lower education, and reduced pregnancy/breastfeeding likelihood), or to both (eg, recurrent depression, suicidal ideation, atypical and circadian subtypes, and other physical/psychiatric conditions). Thirteen of 15 prescription-derived phenotypes were also linked to greater class diversity, strongest for anxiety-related medications (eFigure 4 in Supplement 1). Sex-stratified results are provided for reference noting the imbalance in sample size (9041 female individuals; 3022 male individuals), though effect sizes were concordant (eFigure 5 in Supplement 1; eTable 5 in Supplement 2).

### Genetic Factors Associated With Treatment Complexity

**Figure 2.**
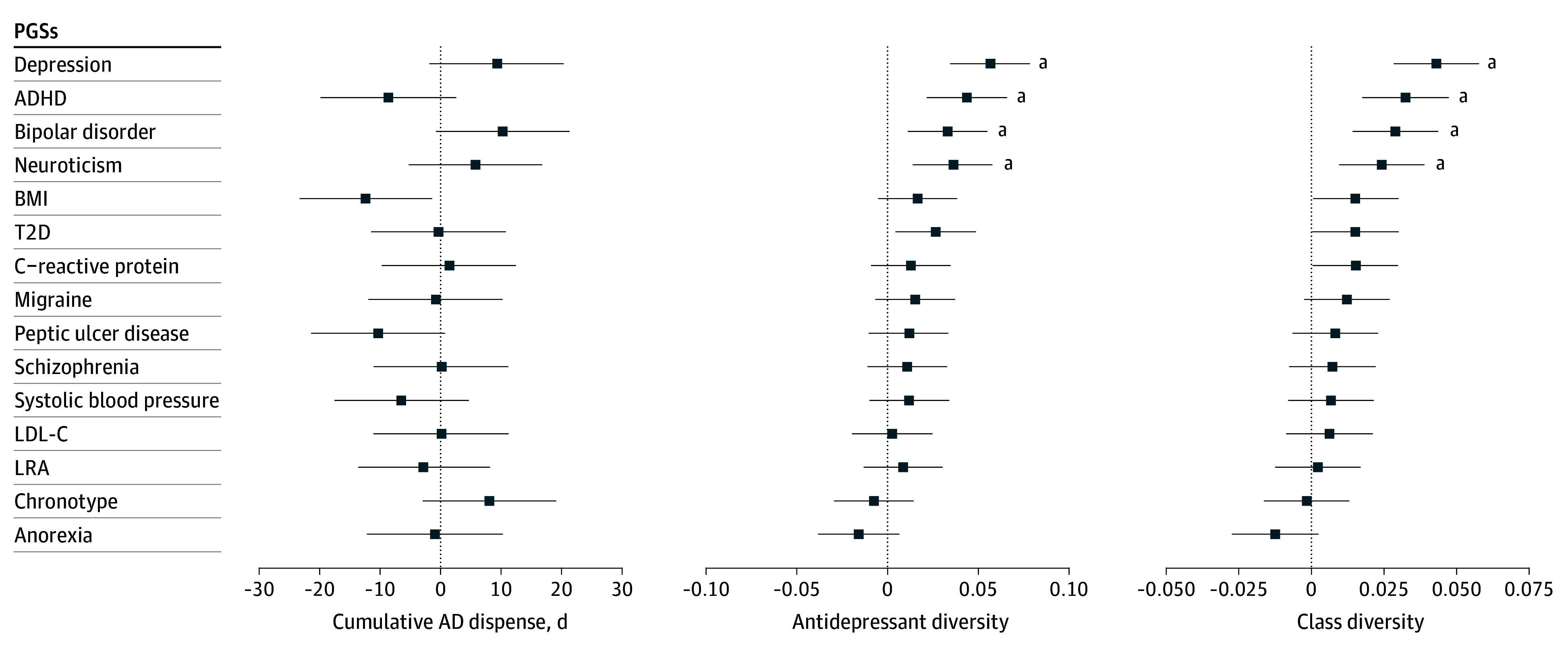
Associations Between Polygenic Scores (PGSs) and Difficult-to-Treat Depression Proxy Metrics PGSs were associated with difficult-to-treat depression proxy metrics among 8898 Australian Genetics of Depression Study (AGDS) participants with lifetime depression. Three difficult-to-treat depression proxy metrics are shown: cumulative all antidepressant (AD) prescription duration (days), AD diversity (range: 1-10), and AD class diversity (range: 1-4). All PGS associations are reported in SD units, standardized across 14 603 AGDS participants of genetically inferred European ancestry. Linear regression models included age, sex, and the first 3 principal components (PCs) as covariates. Complete results, including sensitivity analyses excluding participants with self-reported bipolar disorder, are provided in eTable 6 in Supplement 2. ADHD indicates attention-deficit/hyperactivity disorder; BMI, body mass index; LDL-C, low-density lipoprotein cholesterol; LRA, low relative amplitude; T2D, type 2 diabetes. ^a^Bonferroni correction (corrections applied separately within each metric).

PGSs for 15 traits were tested against treatment metrics (Figure 2; eTable 6 in Supplement 2). While the major depression (MD) PGS, as previously reported,^32^ predicted MDD case status in the AGDS cohort compared to control individuals), it was not associated with cumulative duration among AGDS participants with lifetime MDD but was linked to greater class diversity (β, 0.04 per PGS SD unit; SE, 0.01; *P* = 1.2 × 10^−8^). Psychiatric PGSs for attention-deficit/hyperactivity disorder (ADHD) (β, 0.03; SE, 0.01; *P* = 2.1 × 10^−5^), BIP (β, .029; SE, 0.01; *P* = 1.2 × 10^−4^), and neuroticism (β, 0.02; SE, 0.01; *P* = 1.3 × 10^−3^) were similarly associated with increased class diversity after Bonferroni correction. Results were robust after excluding participants with self-reported BIP (n = 1034) (eFigure 6 in Supplement 1). Sex-stratified analyses are shown in eFigure 7 in Supplement 1 and eTable 7 in Supplement 2.

### Sustained-Use 360 Group Classifications

Among genotyped participants, 5246 (59%) demonstrated sustained-use 360 for a single antidepressant and 5453 (61%) at the class level (eTables 8-11 in Supplement 2): 3149 individuals taking SSRIs, 2020 individuals taking SNRIs, 160 individuals taking TeCAs, and 124 individuals taking TCAs (Table). A total of 821 participants were classified into the BIP−L group, 213 into BIP+L, and 2411 into various. Most (5305 [97.2%]) also met the medication-specific high-continuity criterion (≥360 days, ≤90-day gaps).

**Table.** Key Australian Genetics of Depression Study (AGDS) Participant Summaries Across Class-Level Sustained Antidepressant Use Groups^a^

Variable	Drug class						
	SSRI	SNRI	TCA	TeCA	BIP+L	BIP−L	Various
No.	3149	2020	124	160	213	821	2411
Cumulative AD dispense, median (IQR), d^b^	1031 (685-1331)	1230 (812-1459)	749 (480-1007)	944 (645-1345)	983 (418-1424)	977 (503-1384)	299 (150-890)
AD diversity (range: 1-10), median (IQR)	1 (1-2)	1 (1-2)	1 (1-2)	1 (1-2)	2 (1-3)	2 (1-2)	2 (1-3)
Class diversity (range: 1-4), median (IQR)	1 (1-2)	1 (1-2)	1 (1-2)	1 (1-2)	2 (1-2)	1 (1-2)	2 (1-2)
General adherence score, mean (SD)^c^	1.0 (0.0)	1.0 (0.0)	1.0 (0.1)	1.0 (0.0)	1.0 (0.1)	1.0 (0.1)	0.9 (0.1)
AD-specific high-continuity (≥360 d, ≤90-d gaps), No. (%)	3054 (97)	1978 (98)	118 (95)	155 (97)	NA	NA	NA
AD-specific single continuous prescription episode, No. (%)^b^	1987 (66)	1389 (72)	71 (57)	114 (71)	NA	NA	NA
AD-specific prescription episode duration, median (IQR), d^b^	840 (537-1621)	1196 (632-1635)	735 (465-1571)	999 (534-1625)	NA	NA	NA
AD-specific antipsychotic augmentation, No. (%)	162 (5)	209 (11)	17 (4)	21 (13)	NA	NA	NA
Antipsychotic augmentation with any AD, No. (%)	184 (6.4)	233 (12)	19 (15)	25 (16)	125 (59)	284 (35)	166 (6.9)
Self-reported response to specific AD, No. (%)^d^	2874 (98)	1798 (96)	99 (93)	145 (97)	NA	NA	NA
Self-reported response to any AD, No. (%)^d^	2917 (100)	1844 (100)	103 (100)	153 (100)	181 (100)	742 (100)	1323 (100)
Self-reported all-cause discontinuation of specific AD, No. (%)	175 (9.5)	124 (9.6)	11 (19.4)	13 (11.8)	NA	NA	NA
Self-reported all-cause discontinuation of any AD, No. (%)	925 (100)	736 (100)	51 (100)	67 (100)	108 (100)	455 (100)	853 (100)
Age, mean (SD), y	44 (15)	45 (14)	52 (14)	50 (15)	44 (12)	44 (14)	42 (15)
Sex, No. (%)							
Male	700 (22)	484 (24)	32 (26)	78 (49)	61 (29)	228 (28)	689 (29)
Female	2447 (78)	1534 (76)	92 (74)	82 (51)	151 (71)	591 (72)	1720 (71)
Lifetime MDD score, median (IQR)	8 (7-9)	9 (8-9)	8 (8-9)	9 (8-9)	9 (8-9)	9 (8-9)	8 (8-9)
Age of MDD onset, mean (SD), y	23 (11)	23 (12)	28 (13)	26 (13)	21 (11)	20 (9.8)	22 (12)
No. of MDD episodes, median (IQR)	6 (3-13)	7 (4-13)	6 (3.5-13)	10 (4-13)	13 (6-13)	13 (6-13)	6 (4-13)
Atypical depression subtype, No. (%)	710 (23)	515 (26)	26 (22)	28 (18)	80 (39)	244 (31)	503 (22)
Circadian depression subtype, No. (%)	571 (23)	266 (25)	16 (17)	106 (17)	40 (26)	209 (33)	458 (26)
Suicidal thoughts, No. (%)	2100 (67)	1472 (73)	78 (63)	125 (78)	188 (88)	698 (85)	1694 (70)

Abbreviations: AD, antidepressant; BIP+L, bipolar disorder with sustained lithium treatment; BIP−L, bipolar disorder without sustained lithium treatment; MDD, major depressive disorder; NA, not applicable; SNRI, serotonin-norepinephrine reuptake inhibitor; SSRI, selective serotonin reuptake inhibitors; TCA, tricyclic antidepressants; TeCA, tetracyclic antidepressants.

^a^ All summaries are based on complete cases, with missing values (marked NA) excluded from the denominator, so that prevalence estimates reflect proportions among individuals with available data. Sustained use was defined as 360 or more cumulative days of dispensing. See eTable 9 in Supplement 2 for complete results spanning a range of physical/psychiatric conditions, depression characteristics, and prescription-based proxies for co-occurring conditions.

^b^ Individuals assigned to the combination group (ie, sustained use across multiple classes) were excluded from the total counts of this class-specific summary statistic.

^c^ General adherence score: proportion of adherence interprescription intervals, allowing for clinically appropriate switches and combinations (see the eMethods in Supplement 1 for derivation).

^d^ Treatment response was defined as a binary variable: participants reporting moderately well or very well were classified as responders, and those reporting not at all as nonresponders.

### Treatment Complexity and Prescription Patterns by Medication Class

**Figure 3.**
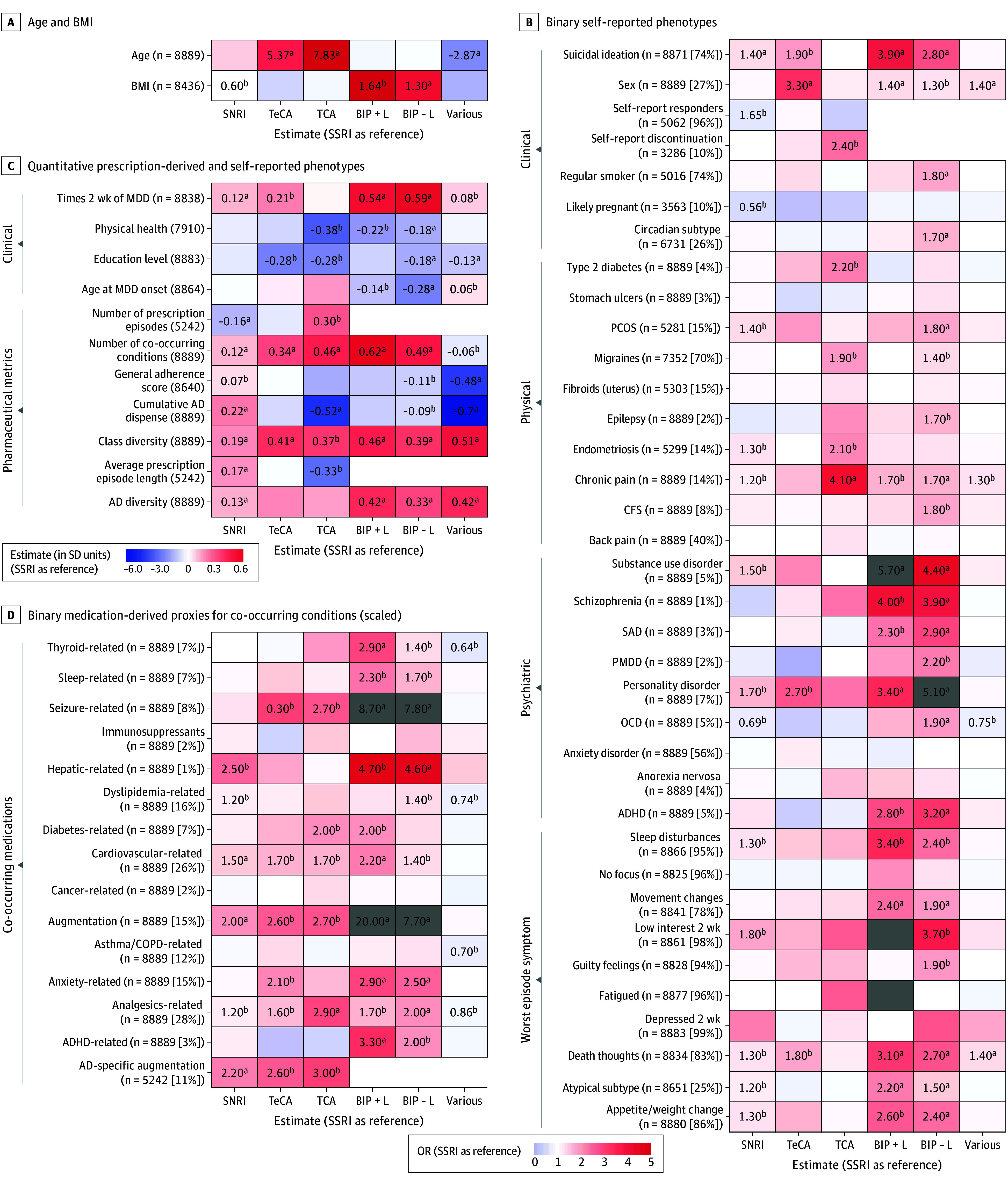
Characteristics Associated With Sustained-Use 360 Class Groups Among Australian Genetics of Depression Study (AGDS) Participants The heatmap shows associations between sustained-use 360 (defined as ≥360 cumulative days of a single antidepressant [AD]) class groups and participant characteristics, with selective serotonin reuptake inhibitor (SSRI) as reference. Sample sizes and trait endorsement percentages are shown on the y-axes in panels C and D. Gray boxes indicate odds ratios greater than 5. All variables except age and body mass index (BMI) were standardized across 12 076 AGDS participants with ≥1 AD dispense. Linear regression models included age and sex as covariates (except when these were outcomes). Higher physical health and education scores indicate better outcomes; female was the reference for sex. AD-specific augmentation refers to augmentation with an antipsychotic. Complete results and sensitivity analyses are in eTables 12-15 in Supplement 2. ADHD indicates attention-deficit/hyperactivity disorder; BIP+L, bipolar disorder with sustained lithium treatment; BIP−L, bipolar disorder without sustained lithium treatment; CFS, chronic fatigue syndrome; COPD, chronic obstructive pulmonary disease; PCOS, polycystic ovary syndrome; PMDD, premenstrual dysphoric disorder; SAD, seasonal affective disorder; SNRI, serotonin-norepinephrine reuptake inhibitor; SSRI, selective serotonin reuptake inhibitors; TCA, tricyclic antidepressants; TeCA, tetracyclic antidepressants. ^a^*P* < .05 after Bonferroni correction (applied separately within each class for 64 tested traits). ^b^*P* < .05 after false discovery rate correction.

Prescription patterns differed by group (Table; statistical comparisons in Figure 3 and eFigure 8 in Supplement 1). General adherence (proportion of adherent inter-prescription intervals, allowing for clinically appropriate switches and combinations) was high (mean = 1.0) across classes except the various group (mean = 0.9). Compared to individuals with sustained SSRI use, all groups showed greater class diversity (β range, 0.19 for SNRIs to 0.51 for various), perhaps indicative of sequential treatment after first-line SSRI. Most had similar or shorter cumulative durations, except SNRIs (longer). Relative to SSRIs (median [IQR] episode, 840 [537-1621] days; 66% single-episode), TCAs had shorter median (IQR) episodes (735 [465-1571] days; 57% single-episode), TeCAs longer (999 [534-1625] days; 71%) and SNRIs even longer (1196 [632-1635] days; 72% single-episode).

### Participant Phenotypes by Medication Class

Sustained-use 360 groups showed distinct phenotypic profiles that remained stable across sensitivity analyses (Figure 3; eResults and eFigures 9-14 in Supplement 1; eTables 12-15 in Supplement 2). After false discovery rate correction, compared to SSRIs, individuals taking SNRIs had higher body mass index (BMI; β, 0.60; SE, 0.2; *P* = 3.4 × 10^−3^), more recurrent MDD, suicidal ideation, atypical depression (OR, 1.2; 95% CI, 1.0-1.4; *P* = 9.3 × 10^−3^), chronic pain, endometriosis, polycystic ovary syndrome, substance use disorder, and personality disorders (OR, 1.7; 95% CI, 1.3-2.1; *P* = 1.5 × 10^−4^), with reduced obsessive-compulsive disorder. Individuals taking TCAs were older, with markedly higher rates of chronic pain (OR, 4.1; 95% CI, 2.8-6.1; *P* = 2.0 × 10^−12^) and endometriosis (OR, 2.1; 95% CI, 1.2-3.6; *P* = 8.9 × 10^−3^), elevated migraines and type 2 diabetes (OR, 2.2; 95% CI, 1.2-4; *P* = 1.2 × 10^−2^), and lower physical health and educational attainment. Individuals taking TeCAs were predominantly older, male, and with higher suicidal ideation (OR, 1.9; 95% CI, 1.3-2.9; *P* = 7.3 × 10^−4^), personality disorders, and lower educational attainment. Across SSRIs, only fluoxetine (vs sertraline) was associated with higher odds of the circadian depression subtype, despite evidence from animal models that many SSRIs show serotonergic modulation of circadian rhythyms.^33^ Relative to SSRIs, all groups had increased augmentation with antipsychotics (ORs, 2.0-20.0) and comedication patterns reflecting their comorbidity profiles (eg, increased analgesic use in those taking TCA).

### Association of PGSs With Medication Class

**Figure 4.**
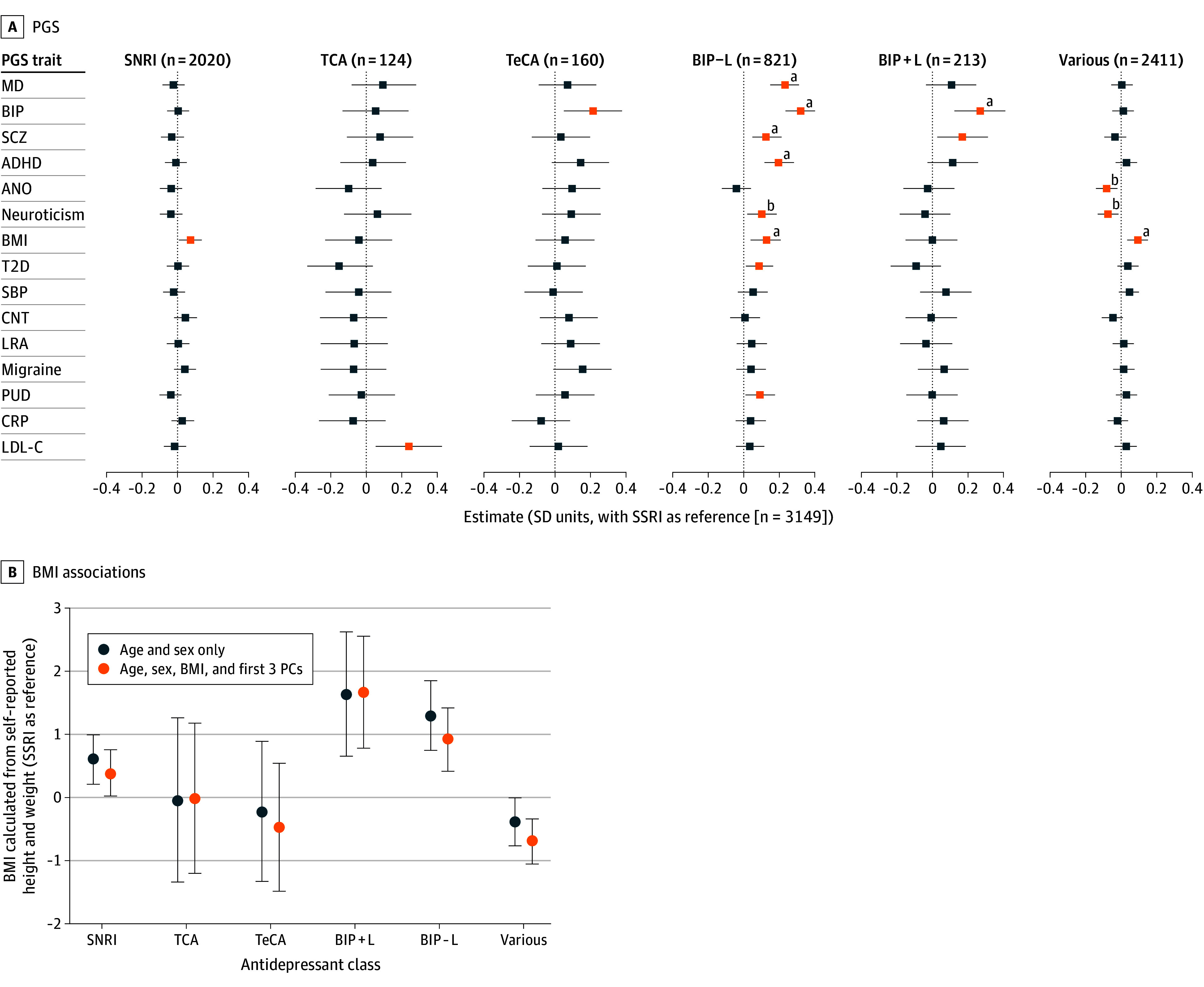
Polygenic and Body Mass Index (BMI) Profiles Across Sustained-Use 360 Class Groups A, Associations between 15 PGSs and class groups, with selective serotonin reuptake inhibitor (SSRI) as reference. All PGSs were standardized (mean [SD], 0 [1]) across 14 603 Australian Genetics of Depression Study (AGDS) participants of genetically inferred European ancestry. Models included the first 3 principal components (PCs) as covariates. B, Associations between self-reported BMI, calculated from self-reported height and weight, and treatment class groups. ADHD indicates attention-deficit/hyperactivity disorder; ANO, anorexia nervosa; BIP, bipolar disorder; BIP+L, bipolar disorder with sustained lithium treatment; BIP−L, bipolar disorder without sustained lithium treatment; BMI, body mass index; CNT, chronotype; CRP, C-reactive protein; LDL-C, low-density lipoprotein cholesterol; LRA, low relative amplitude; MD, depression; PUD, peptic ulcer disease; SBP, systolic blood pressure; SNRI, serotonin-norepinephrine reuptake inhibitor; SSRI, selective serotonin reuptake inhibitors; T2D, type 2 diabetes; TCA, tricyclic antidepressants; TeCA, tetracyclic antidepressants. ^a^*P* < .05 after Bonferroni correction (applied separately within each class for 15 traits). ^b^*P* < .05 after false discovery rate correction.

We next examined associations between antidepressant groups and 15 PGSs (Figure 4A; eTables 16-17 in Supplement 2). The MD PGS did not differ between classes, consistent with MD GWAS cases spanning all classes. As expected, the BIP PGS was significantly higher in BIP+L (β, 0.27 per SD unit of the AGDS cohort; SE, 0.07; *P* = 1.3 × 10^−4^) and BIP−L (β, 0.32; SE, 0.04; *P* = 4.6 × 10^−16^) vs SSRIs. BIP−L also had higher PGSs for MD, schizophrenia, and ADHD, while BIP+L did not. Although BIP−L vs BIP+L differences were nonsignificant, the pattern aligns with reports associating lithium nonresponse with elevated schizophrenia,^12^ MD,^34^ and ADHD^35^ PGSs as well as lithium response to less complex profiles.^36^

Given self-reported BMI differences across classes (Figure 3), additional analyses were conducted to investigate the contributions of genetic predisposition and treatment. Including the BMI PGS as a covariate partially reduced the association of BMI (calculated as weight in kilograms divided by height in meters squared) with the SNRI group compared to the SSRI group from β, 0.60 (SE, 0.20; *P* = 3.4 × 10^−3^) to β, 0.38 (SE, 0.19; *P* = .04) (Figure 4B; eTable 18 in Supplement 2), with similar modest attenuation in the duloxetine and desvenlafaxine subgroups of the SNRI group (eFigure 16 in Supplement 1; eTable 19 in Supplement 2), suggesting both genetic and treatment effects. Duloxetine’s indication for neuropathic pain in Australia^37^ may bias prescribing toward patients predisposed to metabolic disorders. However, mediation analysis indicated that antidepressant class (SSRI vs SNRI) explained only 0.21% (95% CI, 0.01-0.63) of the BMI PGS effect on BMI, with 99.7% due to direct genetic effects (eTable 20 in Supplement 2). As BMI was only self-reported postprescription, interpretation of the indirect effect is limited (eMethods in Supplement 1).

### GWAS on SSRI and SSRI/SNRI Sustained-Use 360 and Self-Reported Efficacy

Since SSRIs were the contemporary first-line treatment in Australia, a GWAS comparing individuals with sustained SSRI use (n = 3022) to those with nonsustained use (n = 4112) was conducted. One independent genome-wide significant locus was identified with top SNV rs4656934, in intron 2 of *SLAMF3* (also known as *LY9*), with the G allele (frequency = 0.31) being associated with reduced odds of sustained SSRI use (OR, 0.81; 95% CI, 0.75-0.87; *P* = 3.5 × 10^−8^) (eFigures 17-18 in Supplement 1; eTable 21 in Supplement 2). *SLAMF3/LY9* encodes a cell surface receptor (protein CD299) involved in the activation of lymphoid cell (B, T, and NK cells)^38^ and is highly expressed in lymphoid tissues (protein atlas).^39^ However, the regulatory architecture of rs4656934 is complex, functioning as both an expression QTL and splicing QTL, with tissue-dependent effects on multiple genes including *CD244*, *TSTD1*, *LY9*,* ITLN2*,* CD224*, *NECTIN4*, and *USF1*.^40^ In EBV-transformed lymphocytes, the G allele shows increased splicing efficiency for some *SLAMF3/LY9* transcripts and increased protein expression, making net regulatory effects somewhat unclear. SNVs at the *SLAMF3/LY9* locus retained suggestive significance (lead SNV rs65463: OR, 0.81; 95% CI, 0.75-0.88; *P* = 1.7 × 10^−7^) in the combined SSRI/SNRI GWAS (eFigures 19-20 in Supplement 1; eTable 22 in Supplement 2), and no SNVs passed genome-wide significance. Neither SSRI/SNRI nor SSRI self-reported efficacy GWAS identified genome-wide significant associations (eFigures 21-22 in Supplement 1; eTables 23-24 in Supplement 2).

## Discussion

While prior studies have focused on symptom-based and molecular-based subtyping of MDD,^27,41,42,43,44,45^ this cohort study proposes an alternative framework grounded in real-world prescription data. We used sustained antidepressant use (≥360 cumulative days) as a pragmatic measure to parse the extensive diagnostic (per the *DSM-5*), genetic, and phenotypic heterogeneity^46^ of MDD. Sustained use of the same antidepressant typically reflects both clinical benefit (efficacy) and tolerability (absence of major adverse effects). As such, we interpret sustained antidepressant use as a proxy for long-term treatment acceptability, encompassing both efficacy and tolerability. Consistent with this interpretation, AGDS participants with sustained use reported better treatment response and lower discontinuation due to side effects than those with shorter cumulative treatment durations (Figure 1D-E).

Despite the distinct phenotypic profiles observed across the sustained-use 360 groups (eg, higher BMI in individuals taking SNRIs, suicidality in those taking TeCAs, and type 2 diabetes in those taking TCAs), both psychiatric and nonpsychiatric PGSs were broadly similar across drug classes. In contrast, psychiatric PGSs for depression, ADHD, BIP, and neuroticism were more strongly associated with greater antidepressant class diversity, suggesting that these genetic liabilities represent transdiagnostic biological factors underlying treatment complexity rather than predictors of specific pharmacologic suitability. This pattern aligns with prior evidence associating MD, ADHD, and neuroticism PGSs with treatment resistance.^10,47,48^ The robust association between treatment complexity and the depression PGS (and not just the PGS for other psychiatric diagnoses) supports the biological mediation of complexity rather than mere diagnostic uncertainty. Notably, the BIP PGS remained associated with treatment complexity even after excluding participants with self-reported BIP, implying that genetic risk for BIP may influence treatment resistance independent of diagnostic status. Overall, real-world antidepressant prescription patterns can delineate phenotypically distinct MDD subtypes, while PGSs may help identify patients at greater risk of difficult-to-treat depression.

Unlike PGSs, self-reported traits were associated with both treatment complexity and longer cumulative duration (Figure 3), meaning reported comorbidity and social factors may play a larger role in prolonged treatment than genetic risk. Traits linked to longer cumulative duration but lower class diversity (eg, type 2 diabetes) characterized classical responders, whereas traits linked to higher class diversity but lower cumulative duration (eg, social factors or other comorbid complexity) reflected poor responders. This aligns with prior evidence linking higher education to better antidepressant outcomes,^19,47^ and smoking to reduced efficacy.^49^ Traits associated with both high diversity and longer cumulative duration (eg, suicidality and self-harm) may identify patients at risk of difficult-to-treat depression. Recognizing these clinical profiles could advance a precision psychiatry approach by tailoring interventions (eg, social interventions for poor responders and intensive monitoring for high-risk patients). Collectively, these results reinforce difficult-to-treat depression as a heterogeneous syndrome rather than a discrete diagnostic category.

We also report, to our knowledge, the first genome-wide significant association with sustained SSRI use via prescription data as the sole outcome. One locus implicates immune signaling, with rs4656934 located within *SLAMF3/LY9*, a cell-intrinsic regulator of T cell activation.^50^ Transdiagnostic genetic risk for psychiatric disorders implicates T cell activation in pathogenesis,^51^ and SSRIs have been shown to have both inhibitory and stimulatory effects on T cell proliferation, dependent on context.^52^ Altered *SLAMF3/LY9* function could promote a lymphoid-linked immune subphenotype of depression, or *SLAMF3/LY9* may modify antidepressant-induced changes in T cell function, leading to altered drug acceptability. This suggests immune dysregulation may underlie a biologically distinct subtype of depression marked by preferential sustained treatment. Future in vitro studies should examine how *SLAMF3/LY9* variants affect T cell responses to antidepressants. However, for a complex disorder like MDD, a single SNV (or risk factor) is unlikely to be predictive; however, once identified, multiple risk factors can be combined to enhance predictive accuracy. Of note, rs4656934 is in strong linkage disequilibrium (*r*^2^, 0.97) with a variant linked to reduced response to repetitive transcranial magnetic stimulation in treatment-resistant depression.^53^ The largest GWAS to date of antidepressant response is a meta-analysis of cohorts (including self-report in AGDS) totaling 94 718 SSRI responders and 19 606 nonresponders (mostly self-report efficacy) identified 2 genome-wide significant loci.^25^ One (rs1106260) had a larger effect size in our analysis, but given the sample size was not significant (eTable 25 in Supplement 2).^25^ Confirmation will require both larger cohorts to increase power for this sustained-use 360 phenotype and orthogonal datasets to establish biological validity.

### Limitations

Several limitations in this study should be acknowledged. First, the 4.5-year prescription window and restriction to the top 10 antidepressants do not capture lifetime treatment trajectories. However, follow-up work shows high consistency in group assignments over time.^54^ Second, prescribing reflects clinician judgment and drug indication, not just biology, and may evolve with changing guidelines, potentially introducing selection bias. Replication in large, genotyped cohorts like Lifelines,^55^ UK Biobank,^55^ and iPSYCH^56^ will be essential to validate our findings and distinguish biological from clinical influences on sustained-use 360. Third, coprescribing for co-occurring conditions (eg, anxiety and ADHD) and antipsychotic augmentation were strongly associated with treatment complexity, indicating that complexity may reflect antidepressant use for conditions other than MDD. Conversely, pregnancy and breastfeeding may account for cases of lower complexity due to prescribing restrictions. Fourth, formal assessment of BIP was not available, and only self-report was used. Fifth, although the sustained-use 360 definition was informed by prescribing guidelines, the threshold remains somewhat arbitrary, and shorter sustained-use durations may also capture meaningful acceptability. Sixth, small sample sizes in the TeCA, TCA, and BIP+L groups reduced precision, and PGS performance varied across traits depending on discovery GWAS characteristics (number of hits, sample size, and SNV-based heritability) (eTable 1 in Supplement 2).

## Conclusions

The findings in this study support real-world sustained antidepressant use as a phenotypically informative approach to depression stratification. If these subtypes and difficult-to-treat depression represent biologically meaningful differences, genetic markers from blood or saliva samples at first diagnosis could help predict treatment outcomes. Retrospective associations such as ours would not only guide the design of prospective studies and inform algorithmic decision-support tools, but also inform clinical decision-making, for example, identifying patients likely to follow a more complex course who may benefit from earlier senior clinical (psychiatrist) input. The clinical feasibility of this approach is demonstrated by trials like Personalise Antidepressant Treatment For Unipolar Depression Combining Individual Choices, Risks and Big Data (PETRUSHKA),^57^ which tested a personalized medicine app to support antidepressant selection, which could be further enhanced by inclusion of predictors of antidepressant sustained use and treatment complexity.

## Notes

Supplement 1. eMethods eResults eFigures eReferences

Supplement 2. eTables

Supplement 3. Data sharing statement
