# Supplementary material for: Genetic and Phenotypic Associations With Sustained Antidepressant Use in Major Depressive Disorder
Source: JAMA Psychiatry. 2026 Jan 28;83(3):247–58. doi: 10.1001/jamapsychiatry.2025.4372 (PMC12853290; doi:10.1001/jamapsychiatry.2025.4372)
Supplement: Supplement 1. — eMethods eResults eFigures eReferences [file jamapsychiatry-e254372-s001.pdf]

Supplemental Online Content

Walker A, Mitchell BL, Lin T, et al. Genetic and phenotypic associations with sustained antidepressant use in major depressive disorder. *JAMA Psychiatry*. Published online January 28, 2026. doi:10.1001/jamapsychiatry.2025.4372

eMethods

eResults

eFigures

eReferences

This supplemental material has been provided by the authors to give readers additional information about their work.

© 2026 Walker A et al. *JAMA Psychiatry*.

Methods

Polygenic Score Calculation

As comorbidities and side-effects influence clinician antidepressant selection, we calculated polygenic scores (PGS) to assess genetic liability for 15 traits linked to depression or BIP, or their treatment response (**eTable 1**). PGS were estimated for major depression (MD; defined as MD because the GWAS combined MDD cases with self-report depression), and common comorbid psychiatric and developmental conditions, including attention deficit hyperactivity disorder (ADHD), anorexia nervosa (ANO), bipolar disorder (BIP), schizophrenia (SCZ), obsessive-compulsive disorder (OCD), and neuroticism. Given the frequent occurrence of metabolic syndrome, autoimmune conditions, and migraines in MDD and its treatment, we also calculated PGS for body mass index (BMI), type 2 diabetes (T2D), systolic blood pressure (SBP), migraines, C-reactive protein (CRP) levels, and low-density lipoprotein cholesterol (LDL-c). Additionally, we included PGS for chronotype, as it is associated with MDD[1], and low relative amplitude (LRA) due to the association between circadian rhythms and lithium response in BIP[2,3]. Based on evidence suggesting a causal relationship between MDD and peptic ulcer disease (PUD)[4], we also calculated a PGS for PUD.

PGS were computed using SBayesRC[5] with GCTB software[6], employing a Bayesian multiple regression framework that integrates functional annotations to optimize SNP weights. Input included GWAS summary statistics and an LD matrix calculated from 20,000 randomly selected UK Biobank genetically inferred European individuals covering ~7.4 million SNPs. Using PLINK2[7], we multiplied joint effect estimates by imputed best guess genotypes and summed values to create PGS, standardized (mean=0, SD=1) across all 14,603 genetically inferred European ancestry participants.

Antidepressant Treatment Complexity Analyses

Before group classification, we examined associations between 44 self-reported traits, 15 prescription-derived proxies for co-occurring conditions, and 15 PGS with three treatment metrics, reflecting components of difficult-to-treat depression (DTD). The analysis included 12,076 participants for prescription-derived and self-reported phenotypes and 8,898 participants with additional genome-wide genetic data of inferred European ancestry for PGS analyses. Treatment complexity was assessed using three metrics based on antidepressant prescriptions: (1) cumulative duration (cumulative days across all 10 antidepressants); (2) medication diversity (number of unique antidepressants tried, range: 1-10); and (3) class diversity (number of unique antidepressant classes tried, range: 1-4). Linear regression models included age at baseline and sex as covariates, and additionally the first three principal components for the PGS analyses. Nominal statistical significance was defined as $p < 0.05$. Given correlation between tests, results are reported significant based on an FDR and more stringently after Bonferroni correction, applied separately across each complexity metric, separately for the PGS and non-PGS analyses. Sensitivity analyses excluded participants with self-reported bipolar disorder (BIP) to account for diagnostic uncertainty when PGS was the outcome, resulting in a final analytic sample of 7,864 participants. As a secondary analyses, sex-stratified associations were undertaken for the 15 PGS traits and the 44-self-reported phenotypes. As a follow-up analyses within the full sample, to investigate the shared polygenic

© 2026 Walker A et al. *JAMA Psychiatry*.

liability for DTD, we associated the three DTD metrics with a weighted PGS, combining PGS significantly associated with the class diversity outcome. This weighted combined score was calculated by taking a weighted sum of the significant PGS (weighted by the square root of each PGS's SNP-based heritability listed in eTable1).

Treatment Group Comparisons

Linear and logistic regression models were used to test differences across treatment groups at drug and class levels. Effect sizes are reported as beta coefficients (+/- SE) for linear models and odds ratios (with 95% CI) for logistic models. All self-report quantitative variables except age and BMI were standardised (mean = 0, SD =1) across the entire AGDS cohort with ≥1 antidepressant dispensed and lifetime depression (N = 12,076). Models were specified as: PGS ~ PC1 + PC2 + PC3 + Treatment_Group, and Outcome ~ Age + Sex + Treatment_Group. We modelled PGS or Outcome ~ Treatment_Group because our research question is descriptive ("Do patients sustaining different antidepressants have different genetic profiles?") rather than predictive ("Can genetics predict treatment choice?"). This approach directly quantifies genetic differences between established treatment groups, with beta coefficients representing mean differences in genetic liability in standardized units.

To obtain all pairwise comparisons between antidepressants (as a results resource in the supplementary material each drug was systematically used as the reference group in turn. However, for consistency in visualisations, we report associations from models using the SSRI group (for class-level comparisons) and the sertraline group (for drug-level comparisons) as the reference groups, given they are the most commonly sustained-use360 treatments in the study. Results are reported significant based on FDR and more stringently after Bonferroni correction, applied within each treatment group for 64 tested traits. For BMI analyses, additional models included BMI PGS and the first three principal components (PCs) adjustment to assess genetic independence.

Adherence and Treatment Continuity Measures

A continuous general adherence score (range 0-1) was calculated for each participant using the proportion of adherent inter-prescription intervals across all antidepressant dispensing, calculated as the number of adherent intervals divided by total number of intervals. Inter-prescription intervals were defined as the time between consecutive dispenses of any antidepressant medication, with adherent intervals classified as ≤90 days between consecutive dispenses and non-adherent intervals as >90 days between consecutive dispenses.

A sensitivity analysis restricting sustained users to those with high within-medication treatment continuity evaluated medication-specific adherence patterns. High treatment continuity was defined using two concurrent criteria: at least 1 prescription episode with ≥360 days total antidepressant dispensing of the same antidepressant, with no individual gap >90 days between consecutive prescriptions. Low treatment continuity included all other dispensing patterns, encompassing participants with substantial treatment gaps, brief treatment episodes, or sporadic medication use. It is important to note that applying this high adherence criterion reduces the number of participants classified as "Combination" users, since many who were exposed to multiple antidepressants do not meet continuity requirements for ≥ 2 drugs. These participants are instead reclassified into the single

© 2026 Walker A et al. *JAMA Psychiatry*.

sustained or various groups, depending on their remaining exposure pattern. This meant that the medication-specific high-continuity requirement could result in a net gain in participants within the single sustained antidepressant-use groups.

Co-prescription Analysis

Select co-prescribed medications were systematically categorized based on primary therapeutic indication and anatomical therapeutic chemical (ATC) classification codes to serve as proxies for specific co-occurring conditions (complete medication list in **eTable 2**).

Co-prescription was defined as ≥3 dispenses of the same medication occurring within the participant's antidepressant exposure window. The exposure window was defined as the period from the first antidepressant dispense date to the last antidepressant dispense date plus 30 days (to account for the final prescription duration) within the study period (July 1, 2013 to December 31, 2017). The ≥3 dispense threshold was selected to distinguish sustained co-medication use from brief trials or acute interventions, while ensuring sufficient prescription frequency to indicate ongoing treatment need or therapeutic response. When multiple medications were indicated for the same condition, at least one medication reaching the ≥3 dispense threshold was sufficient to indicate the presence of that co-occurring condition.

Given the specific clinical role of antipsychotics in depression treatment, more stringent temporal overlap criteria were applied for antipsychotic augmentation. The dispense date of an antipsychotic medication (ATC N05A, excluding lithium) must fall within the active treatment period of an antidepressant prescription, calculated from antidepressant dispense date to estimated end date (dispense date + 30 days), with a lookback tolerance of 0-14 days from dispense date to account for prescription timing variations. A minimum of 3 instances of temporal overlap was required to classify as augmentation therapy.

Derivation of Select Self-reported Phenotypes

A range of self-reported phenotypes spanning physical, psychiatric condition, environmental, social factor dimensions and depression symptoms. Some key derived phenotypes included:

- Atypical depression. Assigned to those responding "yes" to sleeping more during episodes and reporting to "gain" weight during episodes. Only those with non-missing data on both variables were included.
- Circadian depression. Assigned to those meeting at least 3 criteria among 6 circadian features (seasonality, social jetlag, evening chronotype, delayed sleep midpoint, hypersomnia, sleep inertia). Only those with non-missing data on at least 3 circadian features a were included.
- Likely pregnant. Assigned to participants who were likely in active reproductive phases (i.e., pregnancy/childbearing period), calculated as the intersection of the study period with the estimated reproductive timeframe (assuming that pregnancy occurs within 2-years of the previous child, start date = date of first pregnancy; end date = date of first pregnancy + (2 years × number of children)).

© 2026 Walker A et al. *JAMA Psychiatry*.

GWAS on SSRI and SNRI Sustained Use and Self-Reported Efficacy

The primary GWAS compared participants with SSRI sustained-use360 (cases, n=3,022) versus those without SSRI sustained-use360 (controls, n = 4,112). Cases were defined as participants assigned to the class-level SSRI sustained-use360 group. Controls included participants not assigned to this treatment group who also had no history of sustained-use360 of any individual SSRI medication in the 4.5-year window and did not self-report BIP. It is important to note that the control group included those assigned to the SNRI, TCA, and TeCA sustained use groups, in addition those with 'Various' patterns. Thus, approximately half of the control group represents individuals who did not sustain any single medication class, which may reflect factors beyond treatment tolerability or efficacy. A secondary analysis focused specifically on sustained SSRI/SNRI use (cases n=4,973, controls n=2,013).

As a complementary approach, we performed GWAS analyses using self-reported efficacy data for 10 commonly prescribed antidepressants. For the SSRI or SNRI efficacy phenotype, cases (n=7,466) self-reported positive response ("Moderately" to "Very well") to at least one SSRI or SNRI medication, while controls (n=806) did not self-report a positive response ("Not at all well") to any SSRI or SNRI but had available efficacy data for at least one of the 10 antidepressants studied. Participants with missing data for all medications were excluded. A secondary analysis examined SSRI-only efficacy (cases n=5,862, controls n=2,410).

All GWAS analyses were performed using PLINK 2.0 with adjustment for age, sex, and the first three genetic principal components. Only participants of genetically-inferred European ancestry were included, and individuals related at the second-degree or closer were removed (PLINK 2.0 --king-cutoff 0.0884). LD clumping identified independent loci using: significance threshold $P < 5\times10^{-8}$, LD threshold $r^2=0.1$, and distance threshold of 250kb (but report all SNPs that surpassed suggestive significance, $p < 5.0 \times 10^{-6}$, in the supplementary material). SNP-based heritability on the observed scale were estimated using SBayesRC with GCTB software using ~7.4 million SNPs.

Mediation Analyses

We conducted mediation analyses to examine whether BMI polygenic scores (PGS) effects on BMI operate through sustained SSRI vs. SNRI antidepressant treatment choice. ACME (Average Causal Mediation Effect) represents the effect of BMI PGS on BMI that is mediated through treatment selection. ADE (Average Direct Effect) represents the direct effect of BMI PGS on BMI independent of treatment selection. Analysis based on 1,000 quasi-Bayesian simulations using the mediation package in R[8]. Models adjusted for age, sex, and first 3 principal components. Sample size: 4,920 participants (European ancestry only).

However, the mediation pathway (BMI PGS → antidepressant class → BMI) can reflect two mechanisms that cannot be definitively distinguished without baseline BMI data: (1) Selection effects: Genetic liability increases baseline BMI, influencing prescribing decisions (e.g., clinicians preferentially prescribing certain classes based on weight-related considerations), creating an indirect genetic-BMI association via treatment choice. (2) Treatment-induced effects: Different antidepressant classes have differential effects on weight during treatment, with genetic liability potentially modulating susceptibility to these changes.

© 2026 Walker A et al. *JAMA Psychiatry*.

Since BMI was measured towards the end of treatment without baseline measurements, significant mediation could indicate either pre-existing BMI differences that influenced prescribing or genuine drug-induced weight changes during treatment. The mediation analysis quantifies how much of the genetic effect on BMI is transmitted via antidepressant class assignment, but the temporal ambiguity limits causal interpretation of the underlying mechanism.

Results

Supplementary Material 1: Participant Self-reported Phenotypes Differentially Associate with Sustained-use360 Treatment Groups

Here, we explored self-reported clinical characteristics and comorbidities associated with sustained-use360 groups with class effect size estimates expressed relative to SSRIs, and drug effect size estimates expressed relative to SSRI-sertraline (**eTables 12-15, eFigures 9-16**).

SNRIs

SNRI users had significantly higher BMI (β = 0.60, SE = 0.20, p = 3.4 × 10^{-3}), a pattern consistent across the individual SNRIs: duloxetine (β = 1.25, SE = 0.40, p = 1.9 × 10^{-3}), desvenlafaxine (β = 1.12, SE = 0.35, p = 1.5 × 10^{-3}), and venlafaxine (β = 0.67, SE = 0.34, p = 0.049), all relative to sertraline. While long-term antidepressant use is linked to weight gain[9-11], large-scale U.S. EHR data have reported only small differences between SSRIs and SNRIs[12]. However, that study also found slightly greater weight gain with duloxetine versus sertraline[12], consistent with our findings. Among SNRIs, duloxetine was associated with the lowest subjective physical health scores (β = -0.16, SE = 0.06, p = 7.5 × 10^{-3}), mirroring its association with higher BMI. SNRI users also reported more MDD symptoms during their worst episodes, including increased rates of appetite/weight change, low interest and atypical subtype of depression.

Consistent with this severity profile, SNRI users had a higher lifetime psychiatric comorbidity burden, particularly for Personality Disorder (PersD; OR = 1.7, CI = 1.3–2.1, p = 1.5 ×10^{-4}) and substance use disorder (SUD; OR = 1.5, CI = 1.1–2.1, p = 0.010), reflecting the well-established co-occurrence of MD, PersD and SUD[13]. However, SNRI users were less likely to self-report comorbid OCD (OR = 0.69, CI = 0.53–0.91), potentially reflecting prescriber patterns favouring SSRIs for OCD[14]. Duloxetine showed strong associations with somatic comorbidities, including chronic fatigue syndrome (OR = 1.9, CI = 1.2–2.9, p = 2.9 × 10^{-3}), and chronic pain (OR = 2.1, CI = 1.5–2.8, p = 4.1 × 10^{-6}), consistent with its use as an analgesic[15] and the broader clinical picture of poorer subjective physical health in this group. Paradoxically, despite having the highest self-report BMI, the duloxetine group showed elevated rates of self-report lifetime anorexia nervosa (AN, OR = 2.1, CI = 1.2-3.7, p = 1.1 x 10^{-2}) relative to sertraline, highlighting the complex nature of eating disorder presentations.

TCAs (Amitriptyline)

TCA users were significantly older (β = 7.8 years, SE = 1.3, p = 5.9 × 10^{-9}) with lower education attainment (β = –0.28, SE = 0.091, p = 2.0 ×10^{-3}) and the lowest physical health scores overall (β = –0.38, SE = 0.096, p = 6.1 ×10^{-5}), despite similar BMI to SSRI users. TCA use showed significantly more somatic conditions, including T2D (OR = 2.2, CI = 1.2–4.0, p = 0.012), chronic pain (OR = 4.1, CI

© 2026 Walker A et al. *JAMA Psychiatry*.

= 2.8–6.1, p = 2.0 × 10^{-12}), migraines (OR = 1.9, CI = 1.2–3.0, p = 0.011), and endometriosis (OR = 2.1, CI = 1.2–3.6, p = 0.0089). This somatic profile is consistent with their use in migraine prevention[16] and neuropathic pain management[17]. Importantly, TCA users did not show elevated psychiatric comorbidities, distinguishing them from other treatment groups.

TeCAs (Mirtazapine)

Similar to the TCA group, relative to the SSRI sustained use group, TeCA users were older (β = 5.37 years, SE = 1.2, p = 6.5 × 10^{-6}) with lower education attainment (β = –0.28, SE = 0.081, p = 4.3 ×10^{-4}). Male overrepresentation was notable in the TeCA group (OR = 3.1, CI = 2.3–4.2, p = 6.6 ×10^{-13}). They generally had higher rates of suicidal ideation (OR = 1.9, CI = 1.3–2.9, p = 7.1 ×10^{-3}) and death thoughts during their worst MDD episode (OR = 1.8, CI = 1.2–2.8, p = 0.0020), likely reflecting mirtazapine's preferential use in more severe or TRD[18], and its faster onset of action compared to SSRIs[19,20]. At the drug-level, mirtazapine users showed increased appetite/weight change (OR = 2.0, CI = 1.2–3.3, p = 6.2 × 10^{-3}), consistent with its known appetite-stimulating effects[19,20] via 5-HT(3) blockade[21]. Although mirtazapine has shown to benefit insomnia via circadian resynchronisation[22], and in reducing suicidality in insomnia patients[23], we found no evidence of increased sleep disturbances during peak depressive episodes. Unlike TCA users, TeCA users showed significantly higher rates of PersD (OR = 2.7, CI = 1.4–5.0, p = 2.0 × 10^{-3}), but did not exhibit the somatic comorbidity pattern seen with TCAs, aligning with evidence that TCAs are less effective in individuals with comorbid PersD[24].

Self-report Bipolar Disorder Groups (BIP+/-L)

Both groups of participants living with bipolar disorder had higher self-reported BMI, with particularly strong associations for the BIP-L group (BIP-L: β=1.30, SE=0.28, p=3.7 x 10^{-6}; BIP+L: β=1.64, SE=0.51, p=1.2 x 10^{-3}). Unlike the BIP+L group, the BIP-L group reported significantly more mood-related symptoms after Bonferroni correction – low-interest (OR = 5.4, CI = 2.0-9.7, p = 1.7 x 10^{-8}), and guilty feelings (OR=1.9, CI=1.3-2.9, p=1.5 x 10^{-3}).

The BIP-L group showed the broadest psychiatric comorbidity profile, with highest odds of PersD (OR = 5.1, CI = 3.9–6.6, p = 1.5 × 10^{-34}), ADHD (OR = 3.2, CI = 2.3-4.4, p = 2.6 x 10^{-12}), seasonal affective disorder (OR = 2.9, CI = 2.0-4.0, p = 2.2 x 10^{-9}), and uniquely, the circadian depression subtype (OR = 1.7, CI = 1.4-2.1, p = 1.6 x 10^{-7}), OCD (OR = 1.9, CI = 1.4-2.5, p = 5.5 x 10^{-6}), and premenstrual dysphoric disorder (PMDD; OR = 2.2, CI = 1.5-3.2, p = 6.8 x 10^{-5}). Whilst also showing association with lower education attainment (β = –0.18, SE = 0.039, p = 5.2 × 10^{-6}) and earlier depression onset (β = –0.28, SE = 0.033, p = 5.0 × 10^{-17}). In contrast, the BIP+L group did not have lower educational attainment, but showed a severe depression profile, with the highest rate of suicidal ideation (OR = 4.2, CI = 2.8–6.4, p = 1.0 × 10^{-11}), consistent with lithium often being reserved for severe depression in BIP and has been associated with reduced suicide risk following recent self-harm[25]. The group also had a more focused comorbidity profile enriched for SUD, schizophrenia, PersD, ADHD, and SAD. These findings suggest that the BIP−L group captures a more heterogenous subset characterised by extensive psychiatric comorbidity and earlier depression onset. Moreover, the narrower comorbidity profile in the BIP+L group is consistent with literature showing that lithium response is associated with fewer comorbidities than with non-response[26].

© 2026 Walker A et al. *JAMA Psychiatry*.

Supplementary Figures

Supplementary Figure 1.

Defining sustained use and antidepressant treatment group classifications. Column 1: Example patterns of antidepressant use over a 4.5-year period. Please note these examples are based on hypothetical dispensing patterns rather than real data. Column 2: Corresponding sustained single-antidepressant use classification. Column 3: Corresponding class-level sustained use allocation (i.e., SSRI, SNRI, TeCA, TCA, BIP-L, BIP+L, Various). To approximate treatment duration using PBS dispensing data, we first applied a standard 30-day duration per dispense based on typical Australian prescribing practices (Step 1). To avoid overestimating treatment duration due to overlapping prescriptions, the per-prescription duration was calculated as the minimum of either (a) the time until the next dispense of the same medication, or (b) 30 days (Step 2). Cumulative duration was then calculated by summing individual prescription durations, allowing for natural gaps in treatment (Step 3). Sustained use was defined as long-term cumulative use (≥360 days) of a single antidepressant. This definition does not account for strict adherence or continuous use. Cases involving intermittent patterns—such as medication cessation and re-initiation or dispensing in sporadic <8-week blocks—may still be classified as "sustained use" if the total cumulative duration exceeds the 360-day threshold over the 4.5-year window (e.g., MDD_1, MDD_3).

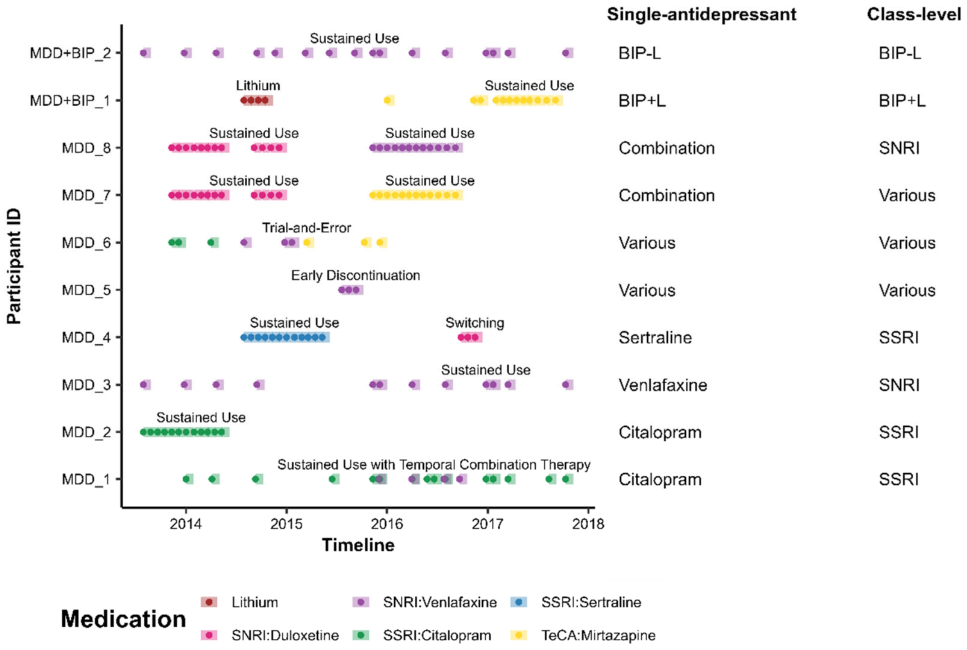

© 2026 Walker A et al. *JAMA Psychiatry*.

Supplementary Figure 2.

Antidepressant use and outcomes in the Australian Genetics of Depression Study (AGDS). Data are shown for 12,074 AGDS participants with lifetime MDD who had ≥1 recorded dispense for one of the 10 most commonly prescribed antidepressants in the cohort, based on linked Pharmaceutical Benefits Scheme (PBS) records from July 2013 to December 2018. These antidepressants are grouped by class (SSRI, SNRI, TeCA, TCA) and summarized across five panels: (A) Number of participants with at least one recorded dispense for each class. (B) Distribution of cumulative prescription duration per antidepressant within each class. The dashed line indicates the threshold used to define sustained antidepressant use (≥ 360 days). (C) Cumulative prescription duration relative to SSRIs (used as the reference class), estimated using linear regression with age and sex as covariates (N = 12,074). The marginal mean for SSRI is shown in black. Classes with statistically significant differences are shown in blue; non-significant differences are shown in grey. (D) Proportion of participants reporting a positive treatment response to an AD, summarised by class stratified by increasing thresholds of cumulative prescription duration (range: ≥1 to ≥1500 days). Responses of "Moderately well" or "Very well" were classified as responders; "Not at all" as non-responders. The dashed line indicates the sustained use threshold. (E) Proportion of participants reporting discontinuation due to any side effects, summarised by class and stratified by cumulative prescription duration (range: ≥1 to ≥1500 days), with the same dashed line indicating the sustained use threshold.

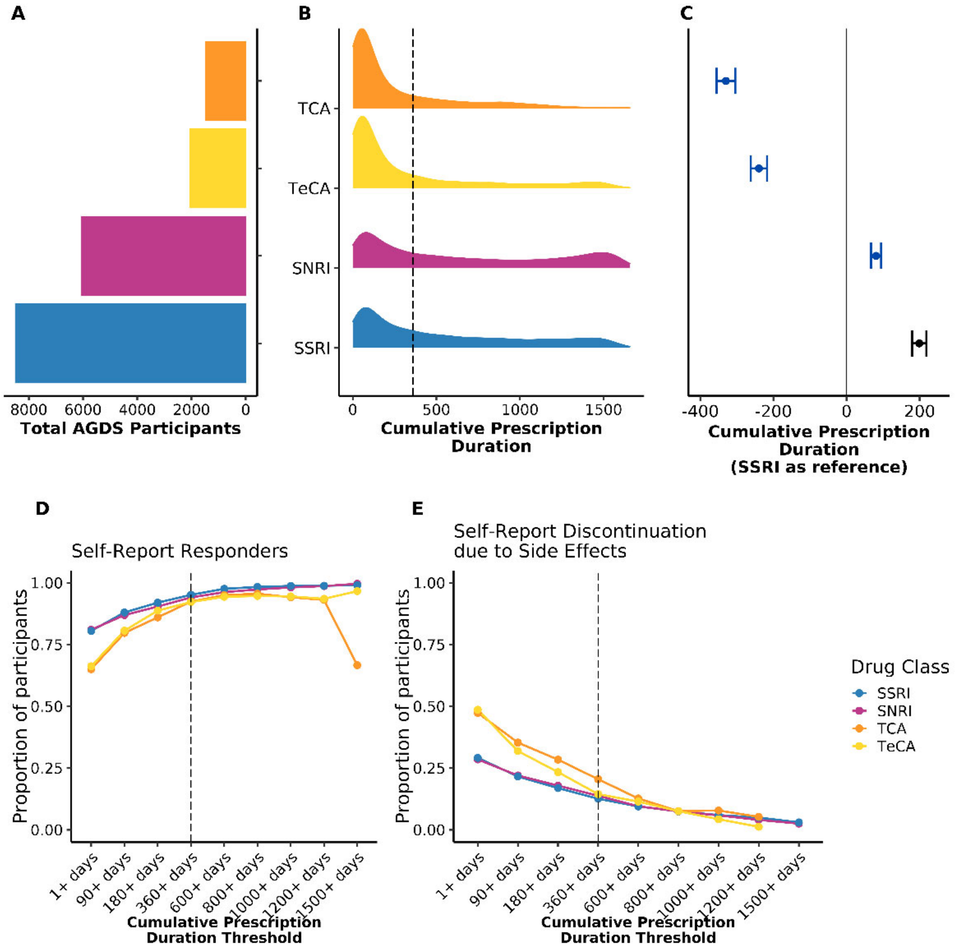

© 2026 Walker A et al. *JAMA Psychiatry*.

Supplementary Figure 3.

Associations between 44 self-reported traits and three antidepressant (AD) use metrics —cumulative AD dispense, medication diversity, and class diversity—among 12,074 AGDS participants. (1) Cumulative all antidepressant prescription duration (days; left panel), (2) AD diversity (range: 1–10; middle panel), (3) Class diversity (range: 1–4, right panel). Models included age and sex as covariates, except when these were the variables of interest. Statistical significance was declared at $p < 0.05$ after false discovery rate (FDR) correction (*) and Bonferroni correction (**), applied separately within each AD use metric for 59 tested traits (44 self-reported and 15 prescription-derived in Supp. Fig. 4). The y-axis reports the number of AGDS participants with complete data for age, sex and the binary trait, as well as the percentage of cases. For interpretation: higher subjective physical health scores reflect better health; higher education levels reflect greater educational attainment; female is the reference group in sex; likely pregnant refers to whether participants were likely in active reproductive phases (i.e., pregnancy/childbearing period), calculated as the intersection of the study period with the estimated reproductive timeframe (date of first pregnancy + 2 years × number of children).

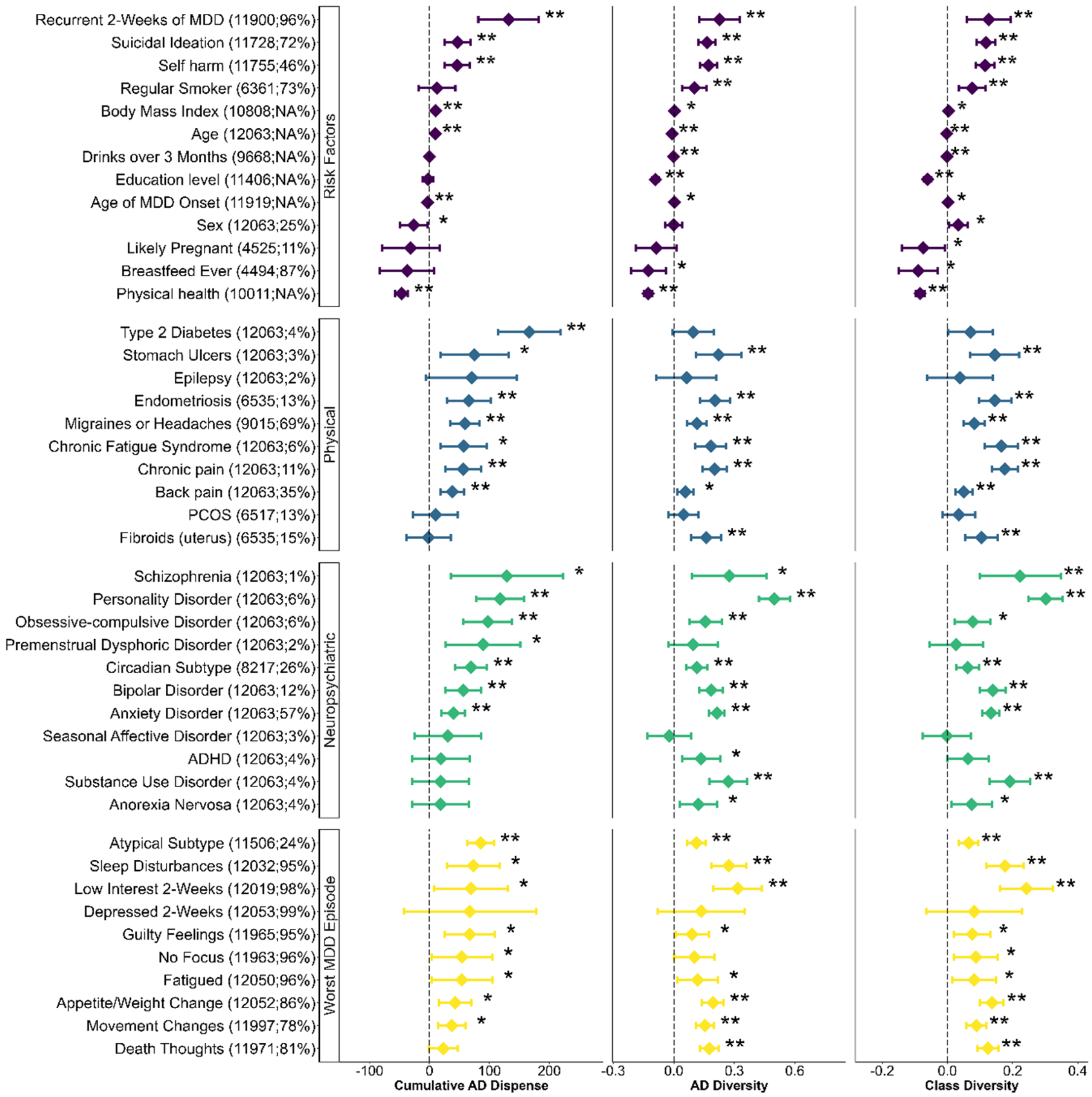

© 2026 Walker A et al. *JAMA Psychiatry*.

Supplementary Figure 4.

Associations between 15 prescription-derived traits and three antidepressant (AD) use metrics —cumulative AD dispense, medication diversity, and class diversity—among 12,074 AGDS participants. (1) Cumulative all antidepressant prescription duration (days; left panel), (2) AD diversity (range: 1–10; middle panel), (3) Class diversity (range: 1–4, right panel). Models included age and sex as covariates, except when these were the variables of interest. Statistical significance was declared at $p < 0.05$ after false discovery rate (FDR) correction (*) and Bonferroni correction (**), applied separately within each AD use metric for 59 tested traits (44 self-reported and 15 prescription-derived). The y-axis reports the number of AGDS participants with complete data for age, sex and the binary trait, as well as the percentage of cases. Detailed derivations of the prescription-based traits are detailed in the supplement and Supp. Table 2.

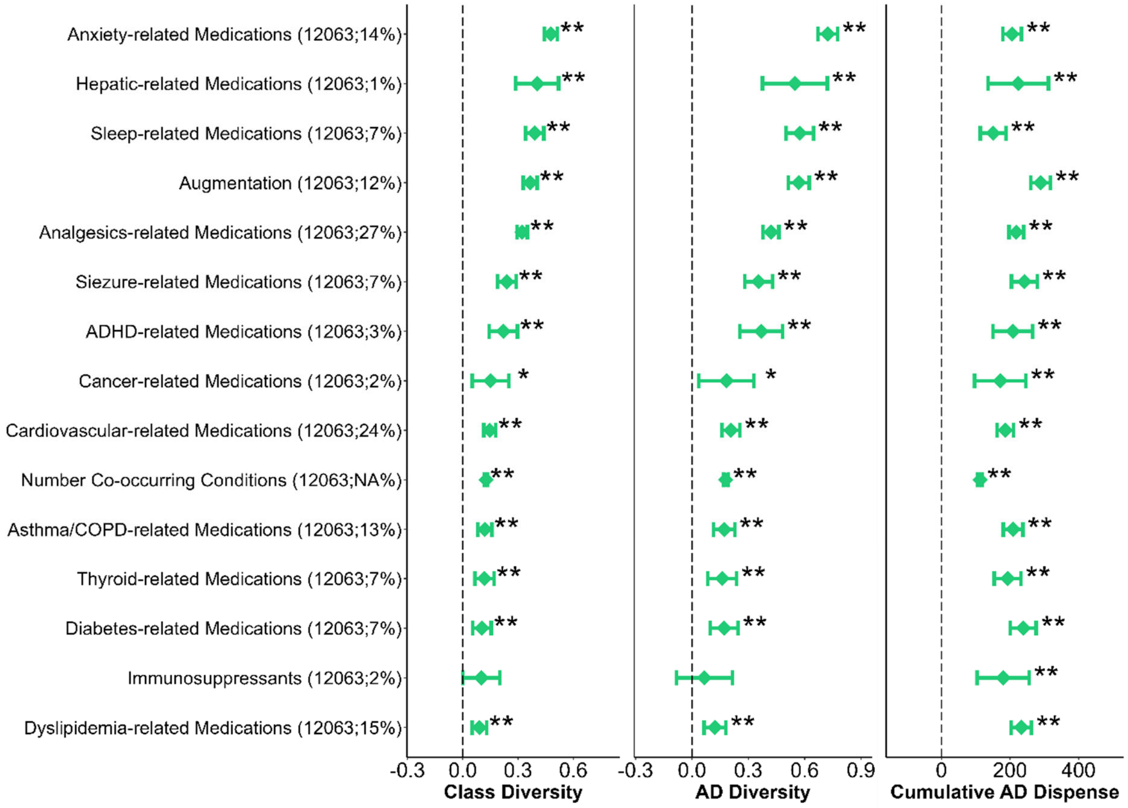

© 2026 Walker A et al. *JAMA Psychiatry*.

Supplementary Figure 5

Sex-stratified associations between 44 self-reported traits and three antidepressant (AD) use metrics — cumulative AD dispense, medication diversity, and class diversity – among 9,041 AGDS Female participants and 3,022 Male participants. (1) Cumulative all antidepressant prescription duration (days; left panel), (2) AD diversity (range: 1–10; middle panel), (3) Class diversity (range: 1–4, right panel). Models included age and sex as covariates, except when these were the variables of interest. Statistical significance was declared at p < 0.05 after false discovery rate (FDR) correction (*) and Bonferroni correction (**), applied separately within each AD use metric-sex stratum combination for 44 tested self-reported traits. The y-axis reports the number of AGDS participants with complete data for age, sex and the binary trait, as well as the percentage of cases. For interpretation: higher subjective physical health scores reflect better health; higher education levels reflect greater educational attainment; female is the reference group in sex; likely pregnant refers to timeframes when participants were likely in active reproductive phases (i.e., pregnancy/childbearing period), calculated as the intersection of the study period with the estimated reproductive timeframe (date of first pregnancy + 2 years × number of children).

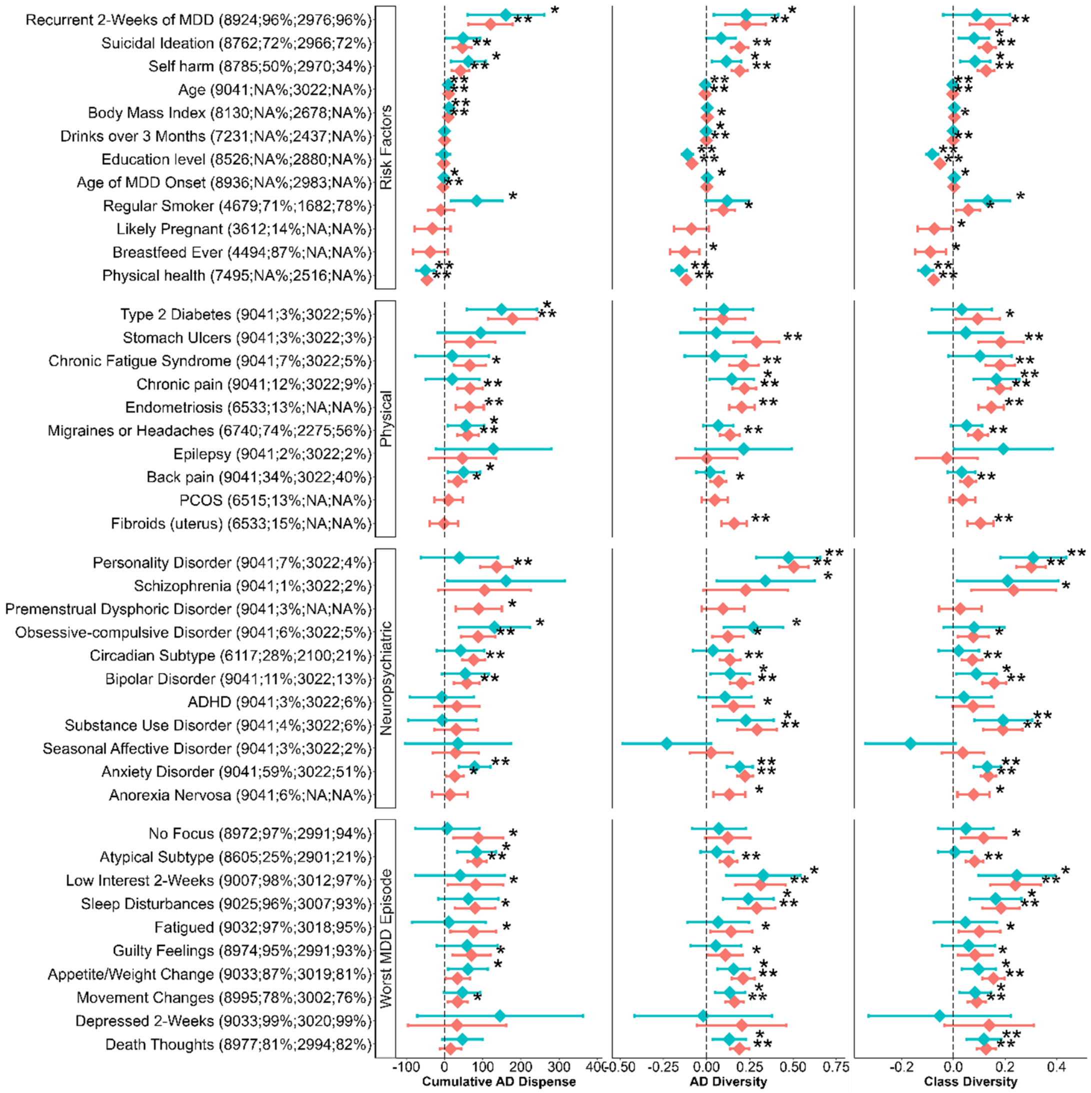

© 2026 Walker A et al. *JAMA Psychiatry*.

Supplementary Figure 6.

Associations between 15 polygenic scores and 3 antidepressant use metrics – cumulative antidepressant dispense, medication diversity, and class diversity - among 7,864 AGDS participants with valid genotyping data and lifetime depression and without self-reported BIP. (1) Left panel: Cumulative all antidepressant prescription duration (days); (2) Middle panel: antidepressant diversity (range: 1–10); (3) Right panel: Antidepressant class diversity (range: 1–4). All PGS associations are reported in standard deviation (SD) units, standardized across 14,603 AGDS participants of genetically inferred European ancestry. Associations were included age, sex, and the first three PCs as covariates. Nominal associations ($p < 0.05$) are represented with a fully coloured circle and the rest with an open circle. Statistical significance was declared at $p < 0.05$ after false discovery rate (FDR) correction (*) and Bonferroni correction (**), applied separately within each antidepressant use metric for 15 tested traits.

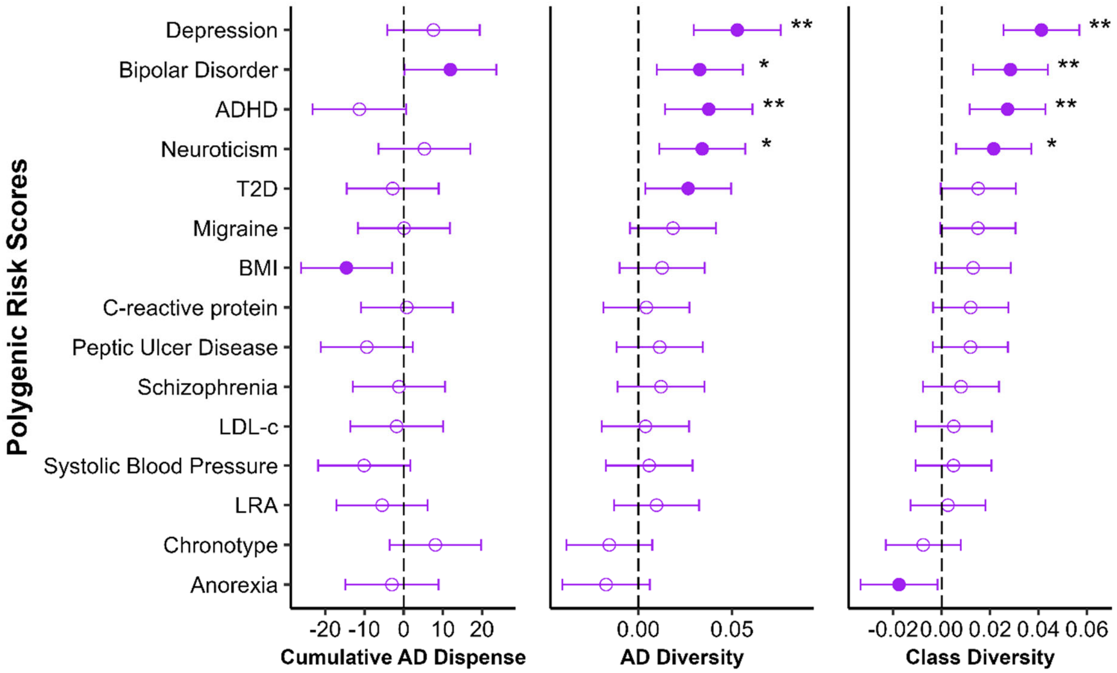

© 2026 Walker A et al. *JAMA Psychiatry*.

Supplementary Figure 7

Sex-stratified associations between 15 polygenic scores and 3 antidepressant use metrics – cumulative antidepressant dispense, medication diversity, and class diversity - among AGDS participants (N=6,617 females, N=2,272 males) with valid genotyping data and lifetime depression. (1) Left panel: Cumulative all antidepressant prescription duration (days); (2) Middle panel: antidepressant diversity (range: 1–10); (3) Right panel: Antidepressant class diversity (range: 1–4). Red estimates represent associations within the female sex and blue estimates represent associations with the male sex. All PGS associations are reported in standard deviation (SD) units, standardized across 14,603 AGDS participants of genetically inferred European ancestry. Associations included age, and the first three PCs as covariates. Nominal associations ($p < 0.05$) are represented with a fully coloured circle and the rest with an open circle. Statistical significance was declared at $p < 0.05$ after false discovery rate (FDR) correction (*) and Bonferroni correction (**), applied separately within each antidepressant use metric for 15 tested traits.

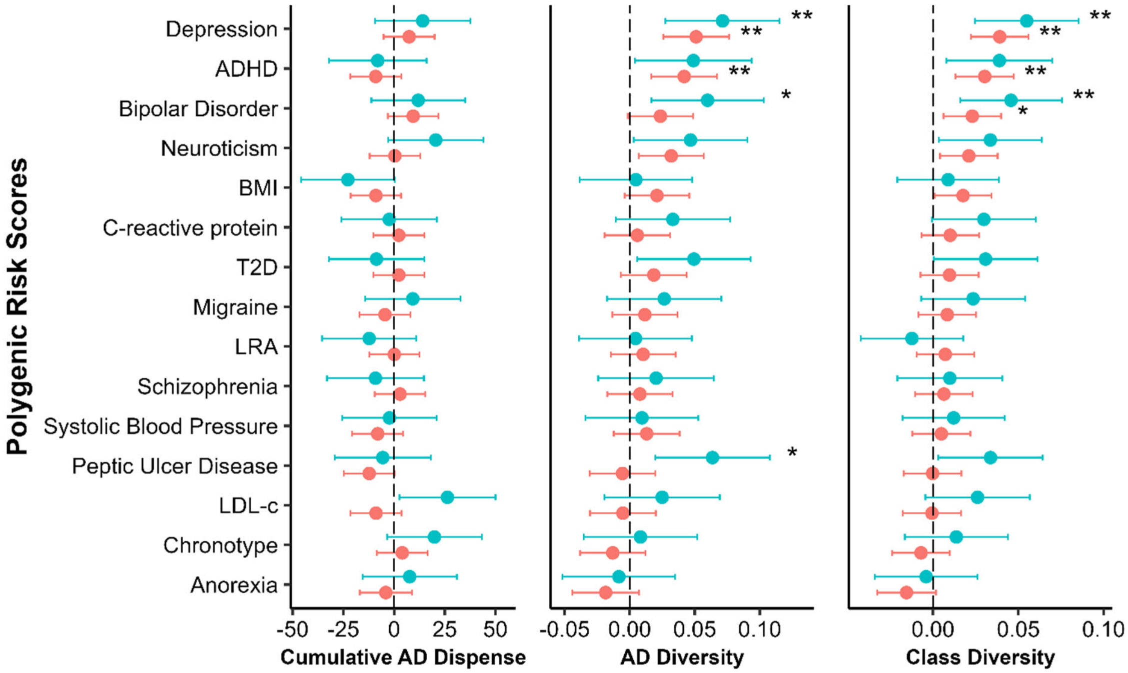

© 2026 Walker A et al. *JAMA Psychiatry*.

Supplementary Figure 8. Heatmap of participant characteristics associated to class-level high-continuity sustained antidepressant use (≥360 days, ≤90-day gaps) groups. Dependent variables associated across treatment groups included: (A) unscaled age and BMI; (B) scaled binary outcomes spanning self-reported phenotypic, physical, psychiatric traits, symptoms during the worst MDD episode; (C) scaled quantitative outcomes related to pharmaceutical metrics and phenotypic feature; (D) scaled binary outcomes related to medication-derived proxies for co-occurring conditions. The y-axis in (C) and (D) reports the number of AGDS participants with complete data for age, sex and the binary trait, as well as % endorsing the trait. Grey boxes indicate odds ratios (OR) > 5. All dependent variables (except age and BMI) were standardized (mean = 0, SD = 1) across the entire AGDS cohort with ≥1 antidepressant (AD) dispense (N = 12,074). All models included age and sex as covariates, except when these variables were the outcomes of interest. The SSRI group served as the reference category. Statistical significance was determined at $p < 0.05$ after correction for multiple testing: false discovery rate (FDR; *) and Bonferroni correction (**), applied separately within each AD class metric for 64 tested phenotypes. For interpretability: higher physical health scores indicate better health; higher education scores indicate greater educational attainment; and "female" was the reference group for sex. The correlation between test statistics under the two continuity thresholds was 0.997.

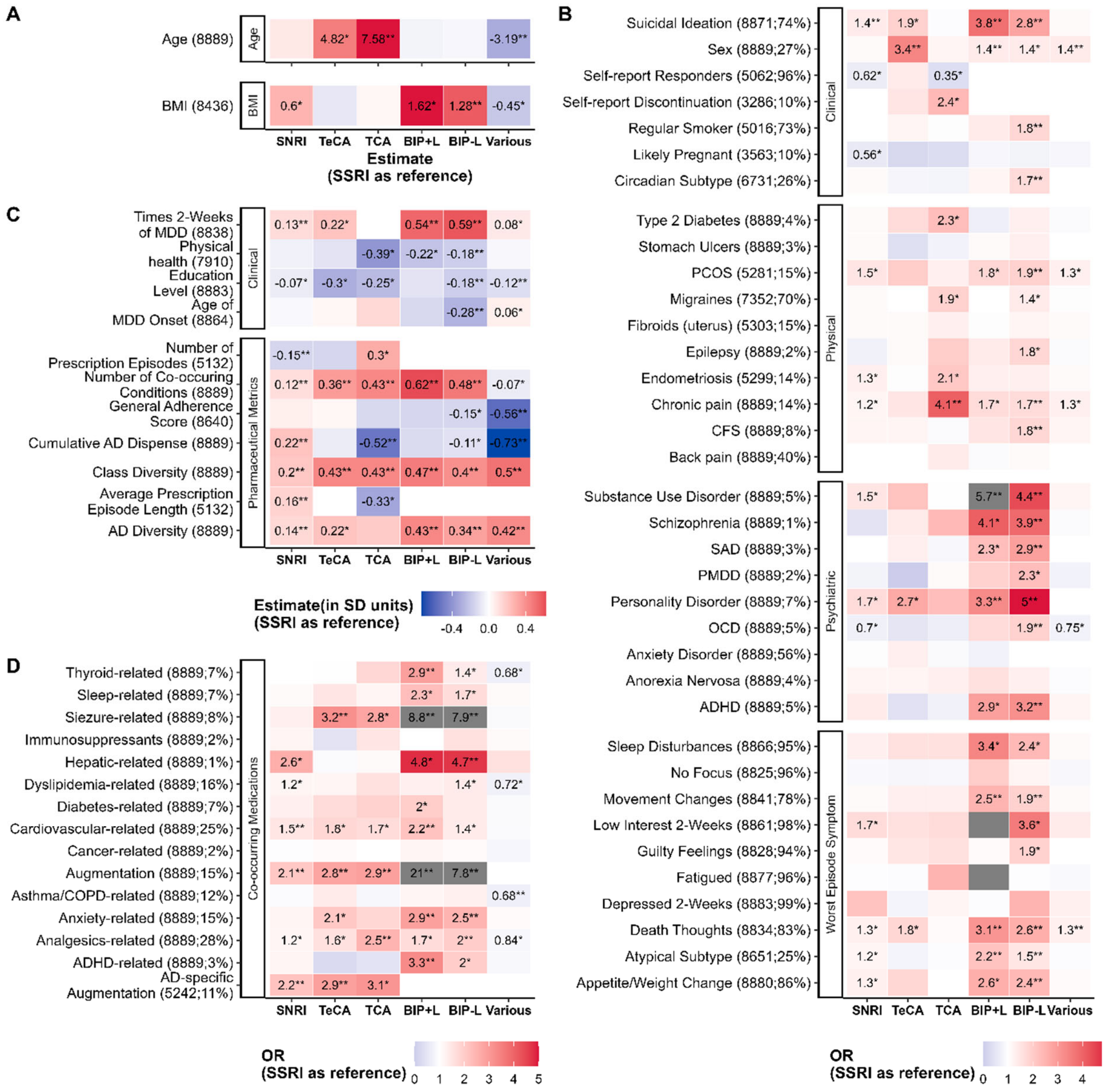

© 2026 Walker A et al. *JAMA Psychiatry*.

Supplementary Figures 9 and 10. Associations between self-reported and prescription-derived quantitative variables with sustained antidepressant use groups under two sustained use thresholds. Analyses were conducted separately under two cumulative dispensing thresholds: (1) ≥360 days and (2) ≥600 days. Supplementary Figure 9 presents associations with pharmaceutical metrics. Supplementary Figure 10 presents associations with clinical features. All variables, except age and BMI, were standardized (mean = 0, SD = 1) across the full AGDS cohort with at least one recorded antidepressant dispense (N = 12,074). Linear models included age (in years) and sex (female as reference) as covariates, except when age was the dependent variable. Statistical significance was defined as $p < 0.05$ following multiple testing correction. Asterisks indicate significance after false discovery rate (FDR) correction (*) and Bonferroni correction (**), applied separately within each threshold–drug group combination for 64 tested phenotypes. The correlation between test statistics under the two minimum sustained use thresholds for pharmaceutical (Fig. 9) and clinical features (Fig. 10) was 0.97 for both.

© 2026 Walker A et al. *JAMA Psychiatry*.

Supplementary Figure 9

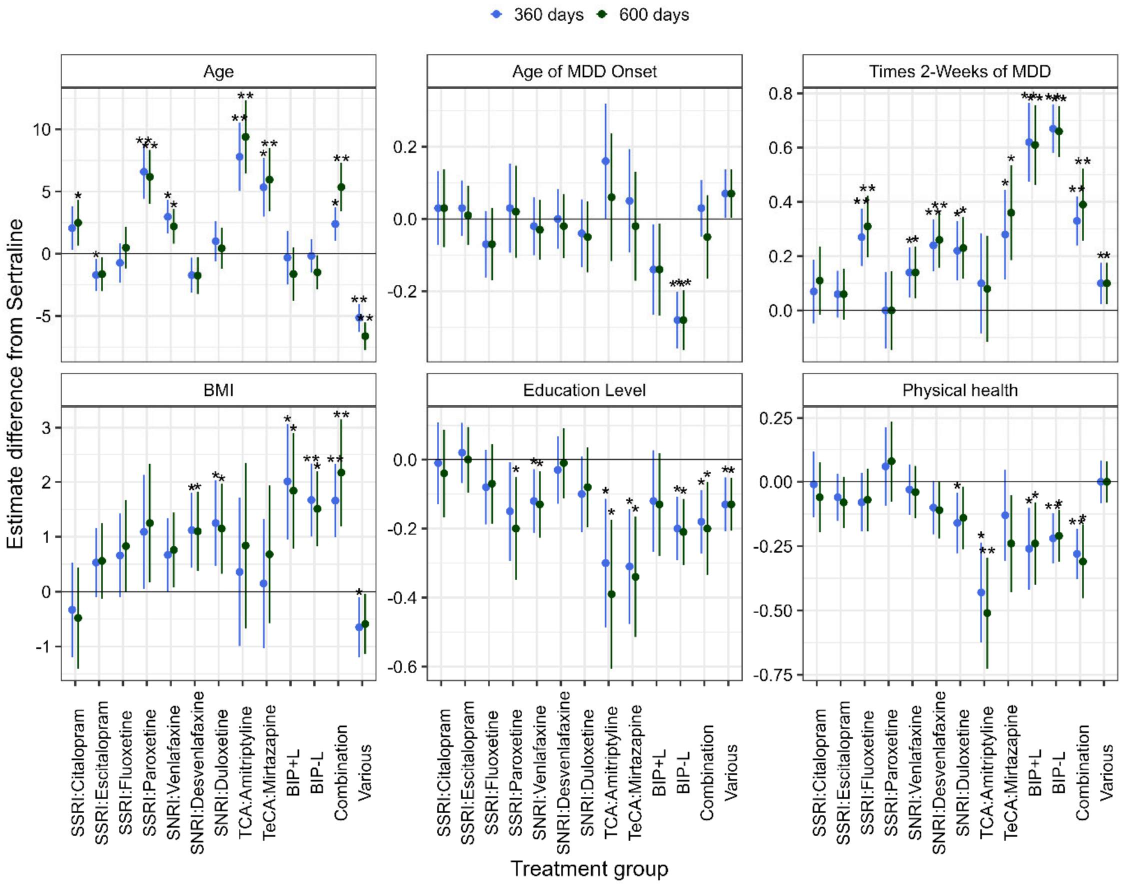

© 2026 Walker A et al. *JAMA Psychiatry*.

Supplementary Figure 10

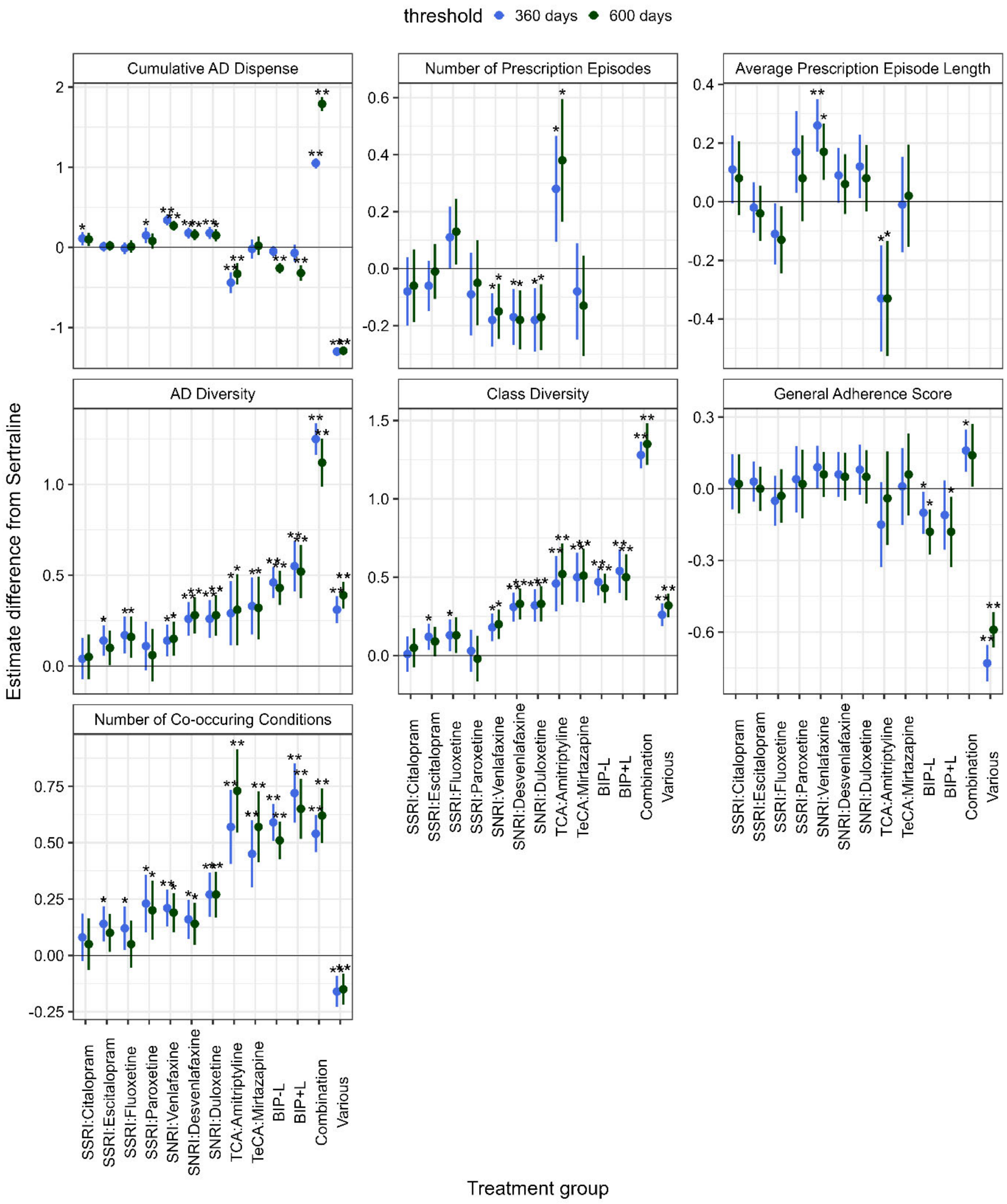

© 2026 Walker A et al. *JAMA Psychiatry*.

Supplementary Figures 11, 12, 13 and 14. Associations between self-reported and prescription-derived binary variables with sustained antidepressant use groups under two sustained use thresholds. Analyses were conducted separately under two cumulative dispensing thresholds: (1) ≥360 days and (2) ≥600 days. Supplementary Figure 11 presents associations with risk factors and treatment response phenotypes. Supplementary Figure 12 presents associations with physical and psychiatric traits. Supplementary Figure 13 presents associations with symptoms reported during the worst MDD episode. Supplementary Figure 14 presents associations with medication-derived proxies for co-occurring conditions. All variables were standardized (mean = 0, SD = 1) across the full AGDS cohort with ≥1 antidepressant dispense (N = 12,074). Models included age (in years) and sex (female as reference) as covariates, except when sex was the outcome. Statistical significance was defined as $p < 0.05$ after false discovery rate (FDR) correction (*) and Bonferroni correction (**), applied separately within each threshold-drug group combination for 64 tested phenotypes. The correlation between test statistics under the two minimum sustained use thresholds for each set of features within the 4 figures were 0.95 (fig. 11), 0.97 (fig. 12), 0.97 (fig. 13), and 0.98 (fig. 14), respectively.

© 2026 Walker A et al. *JAMA Psychiatry*.

Supplementary Figure 11

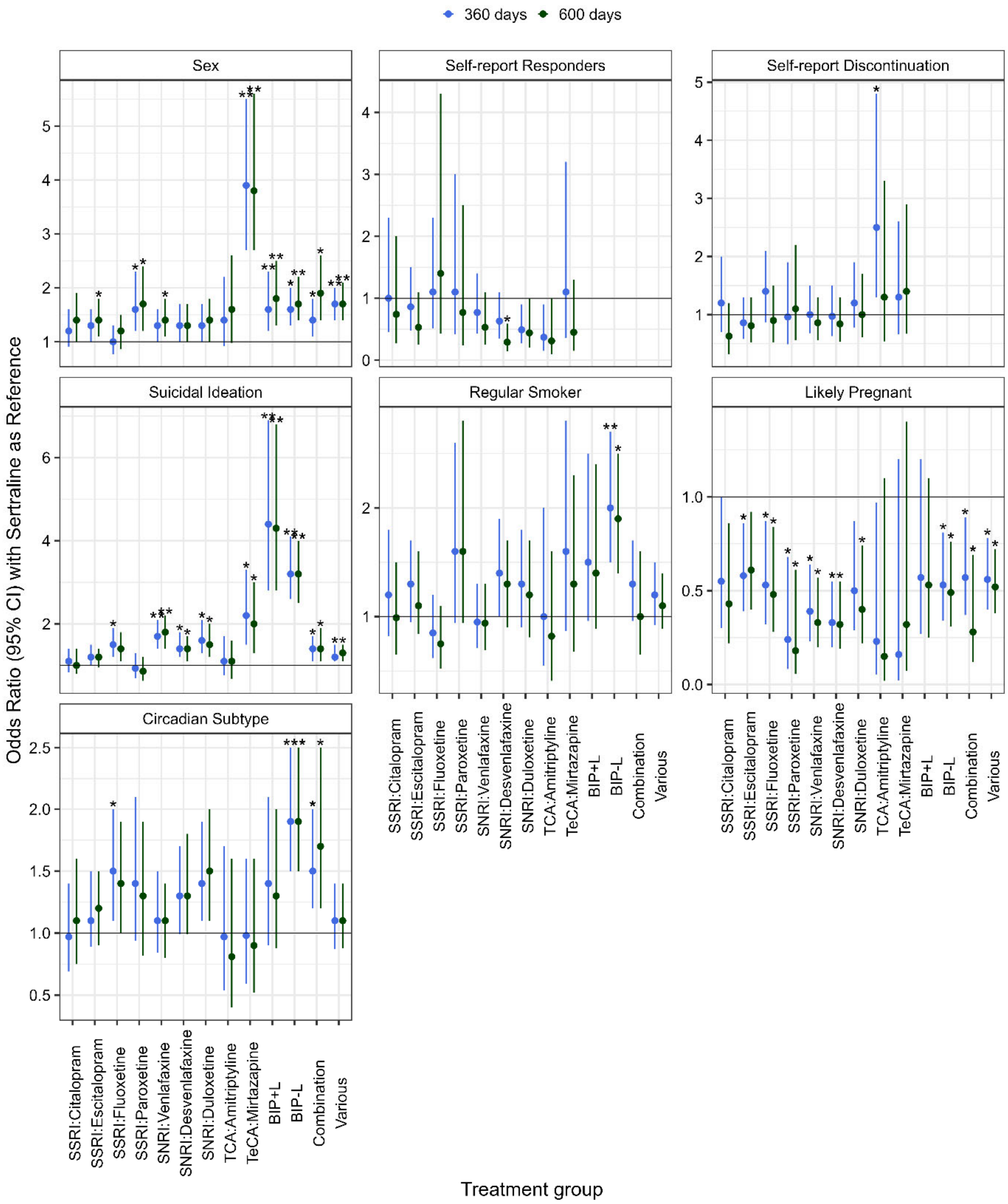

© 2026 Walker A et al. *JAMA Psychiatry*.

Supplementary Figure 12

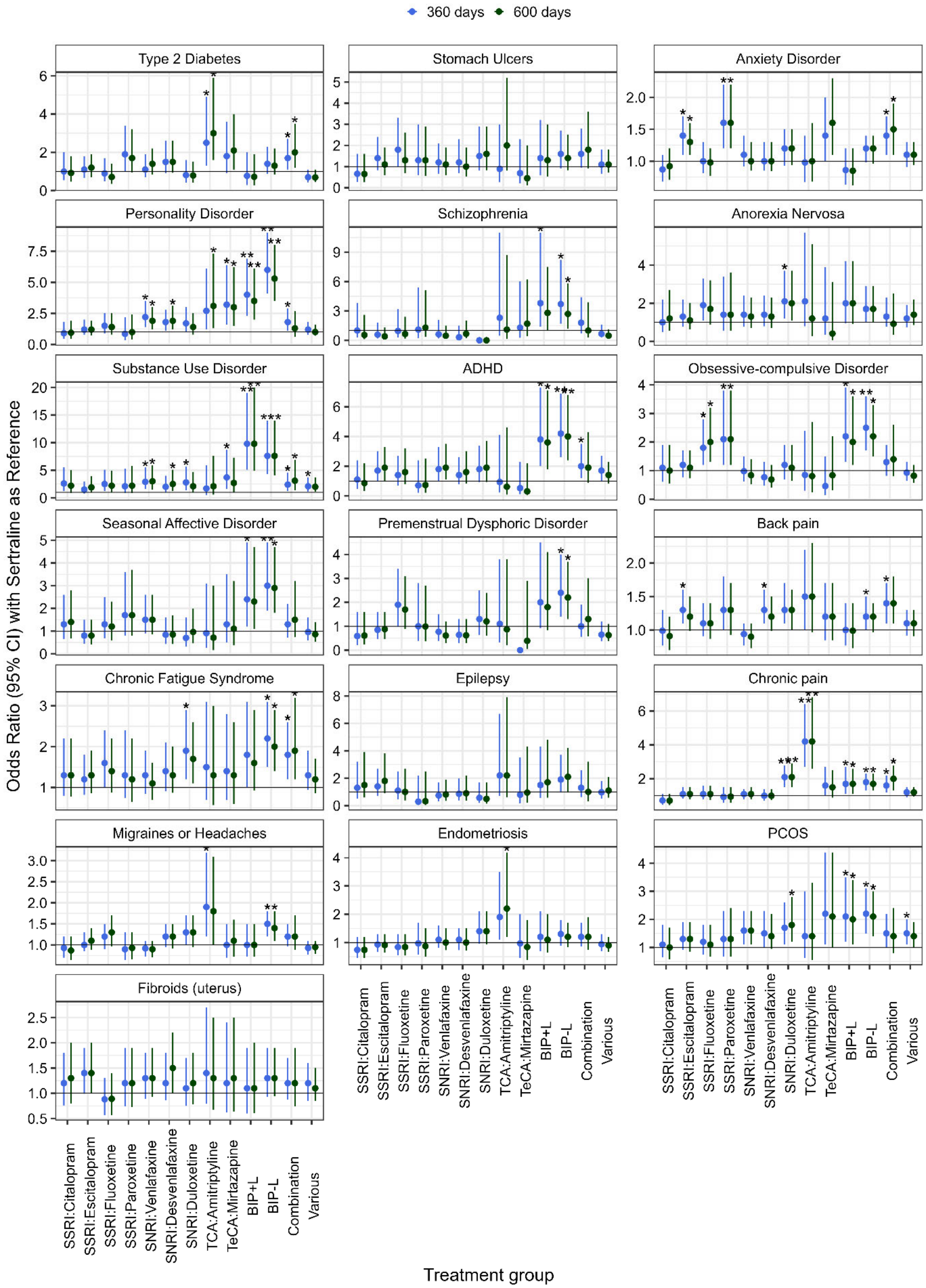

© 2026 Walker A et al. *JAMA Psychiatry*.

Supplementary Figure 13

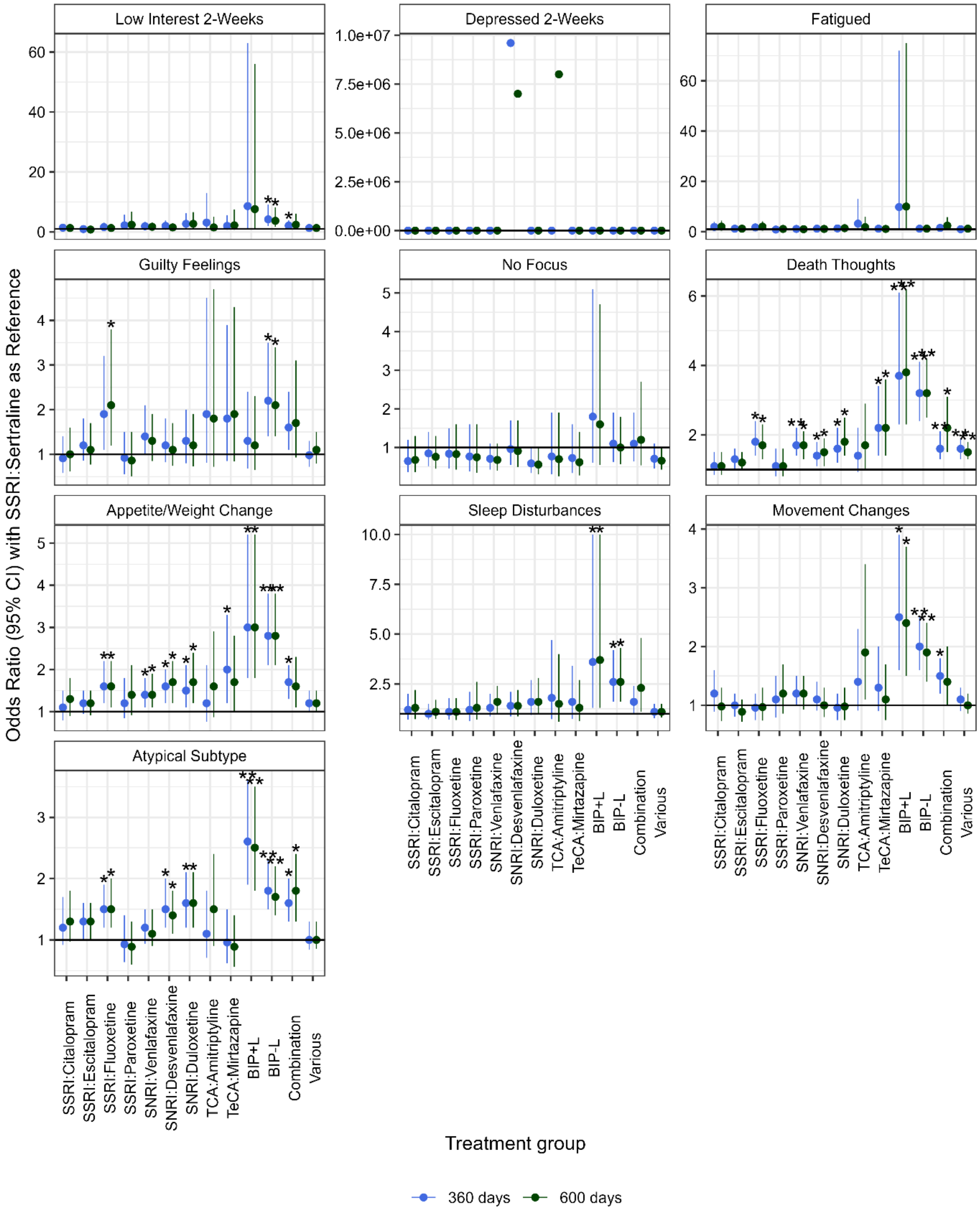

© 2026 Walker A et al. *JAMA Psychiatry*.

Supplementary Figure 14

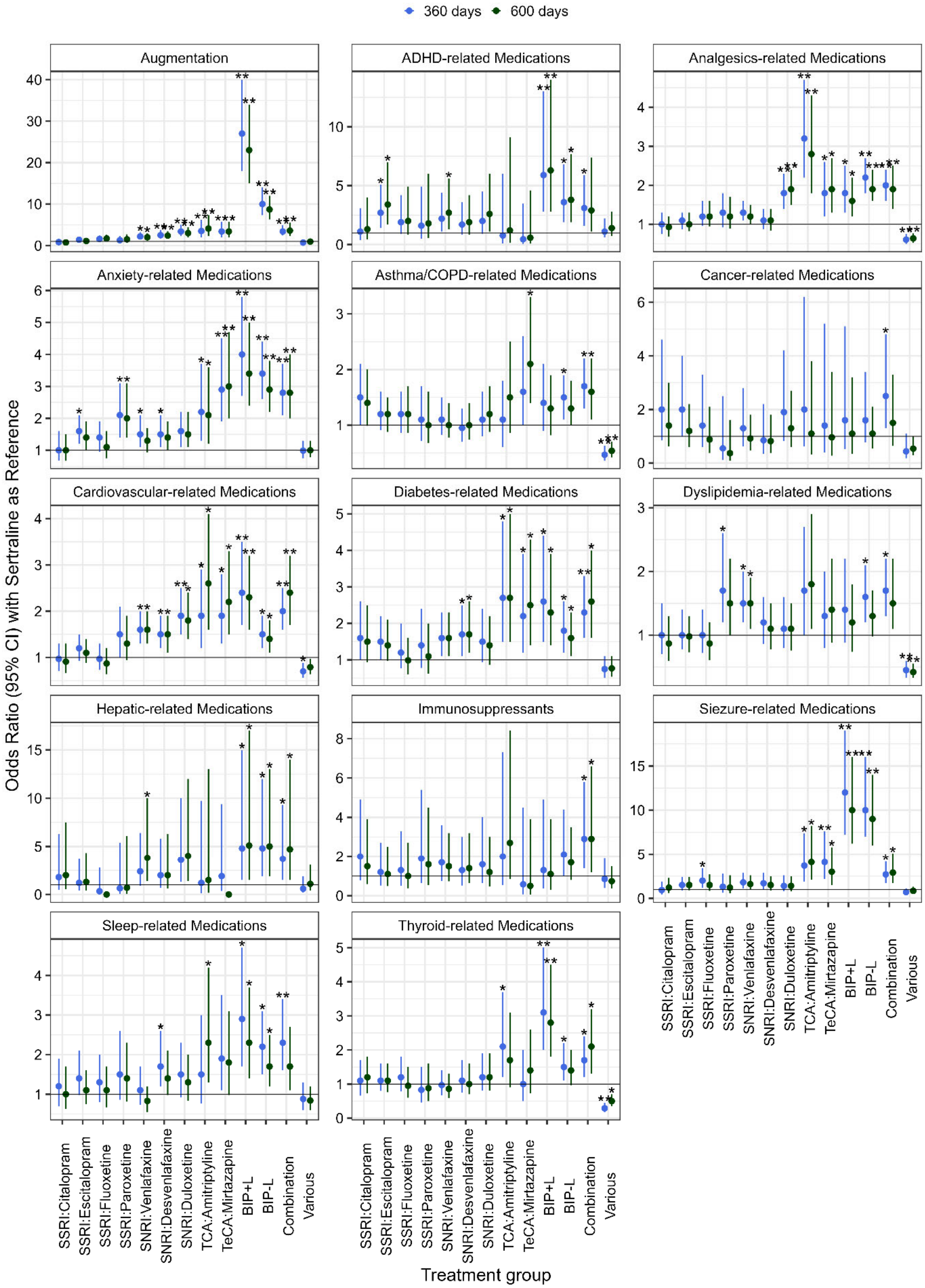

© 2026 Walker A et al. *JAMA Psychiatry*.

Supplementary Figure 15

Associations between polygenic risk scores and antidepressant treatment groups under two sustained use thresholds. Associations between 15 polygenic risk scores (PGS) and drug-level sustained antidepressant use groups were examined separately under two cumulative dispensing thresholds: (1) ≥360 days and (2) ≥600 days. All PGS were standardized across 14,603 AGDS participants of genetically inferred European ancestry. Associations included the first three PCs as covariates. Statistical significance was defined as $p < 0.05$ following multiple testing correction. Asterisks indicate significance after false discovery rate (FDR) correction (*) and Bonferroni correction (**), applied separately within each threshold group for 15 tested PGS traits. The correlation between test statistics under the two minimum sustained use thresholds was 0.91.

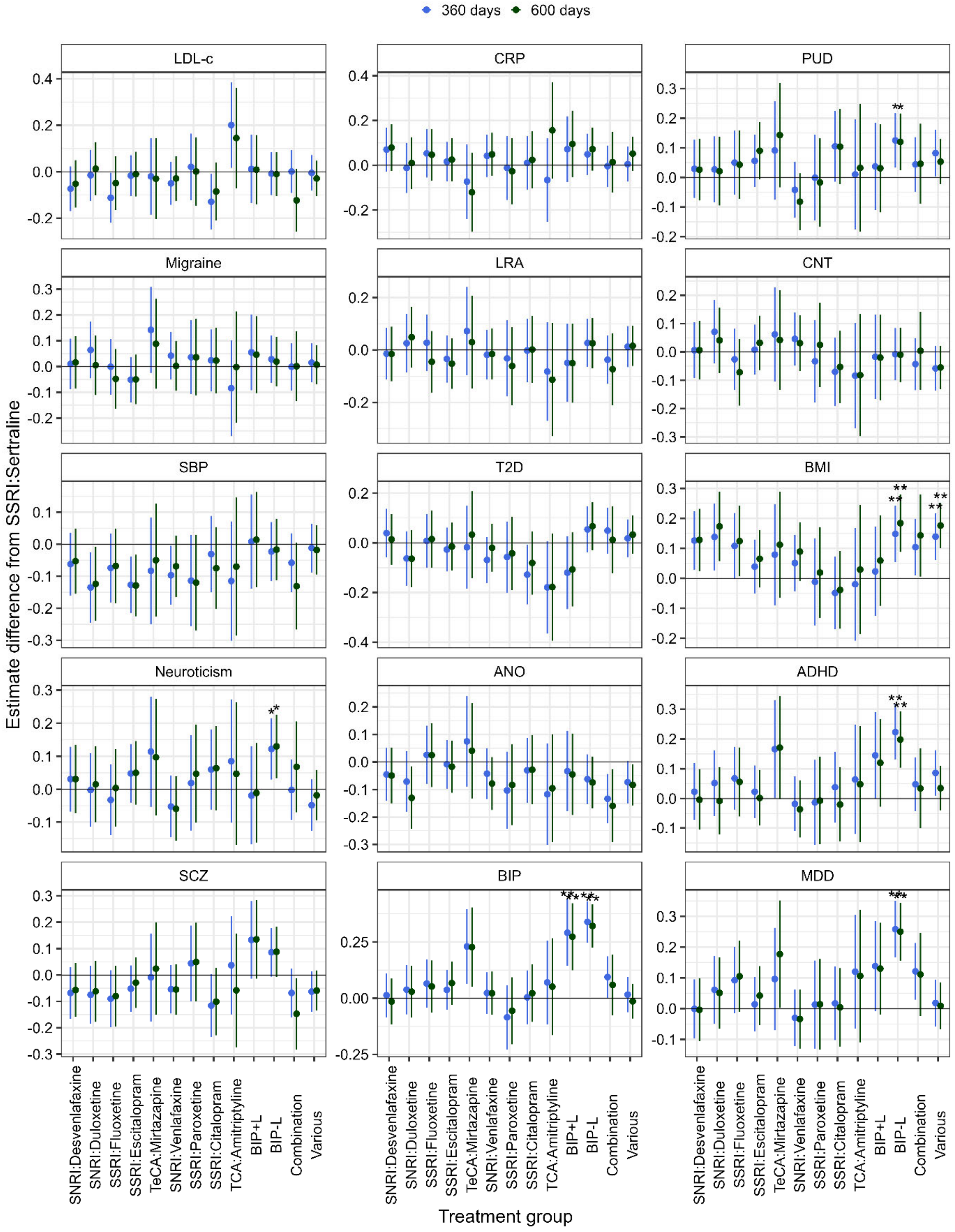

© 2026 Walker A et al. *JAMA Psychiatry*.

Supplementary Figure 16

Association between BMI and BMI adjusted by BMI polygenic score (PGS) with sustained antidepressant use groups under two sustained use thresholds. Body mass index (BMI), calculated from self-reported height and weight, was examined in relation to drug-level antidepressant groups under two cumulative dispensing thresholds: Top panel: ≥360 days; Bottom panel: ≥600 days. Analyses were conducted separately for each threshold group using linear regression models including age (in years), sex (female as reference) as covariates. Associations were run unadjusted by BMI PGS (black markers), and also adjusted by BMI PGS, as well as the first three PCs (blue markers). The PGS was standardized across the full AGDS cohort of genetically inferred European ancestry (N = 14,603). Significance markers were omitted because the single-trait adjusted BMI analysis and the multi-trait phenotypic analysis require different multiple testing approaches, making direct comparison of significance levels inappropriate.

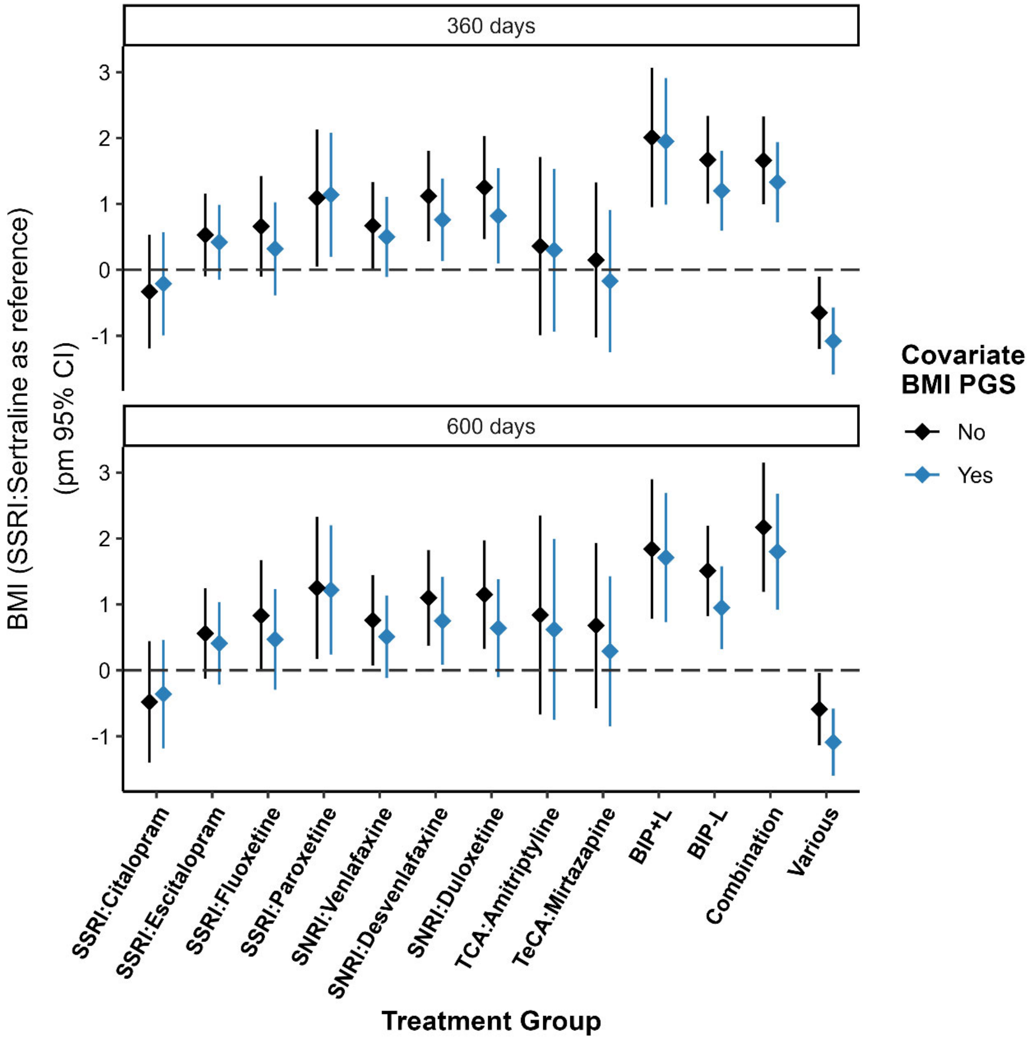

© 2026 Walker A et al. *JAMA Psychiatry*.

Supplementary Figures 17 and 18. Genome-wide association study (GWAS) of sustained SSRI use. A logistic regression GWAS was conducted comparing participants with sustained SSRI use (cases, n = 3,022) to those with no sustained use for any SSRI (controls, n = 4,112). Cases and controls consist of participants without self-reported bipolar disorder. Only participants of genetically inferred European ancestry were included, and individuals related at the second-degree or closer were removed (PLINK 2.0 --king-cutoff 0.0884). Analyses were performed using PLINK 2.0, including age, sex, and the first three genetic principal components (PCs) as covariates. Supplementary Figure 17: Quantile-quantile (QQ) plot and the genomic inflation factor ($\lambda = 1.002$) indicating minimal inflation. Supplementary Figure 18: Manhattan plot of all common SNPs. SNPs surpassing suggestive significance ($p < 5.0 \times 10^{-6}$) are highlighted in green. SNP-based heritability was estimated using SBayesRC in GCTB software based on approximately 7.4 million SNPs. The SNP-based heritability was estimated at $h^2 = 0.15 \pm 0.106$ (mean ± SD), based on an observed phenotypic variance of 0.24 for the binary sustained SSRI/SNRI use trait. The wide uncertainty reflects known limitations in heritability estimation for binary traits in moderately sized samples[27].

Supplementary Figure 17

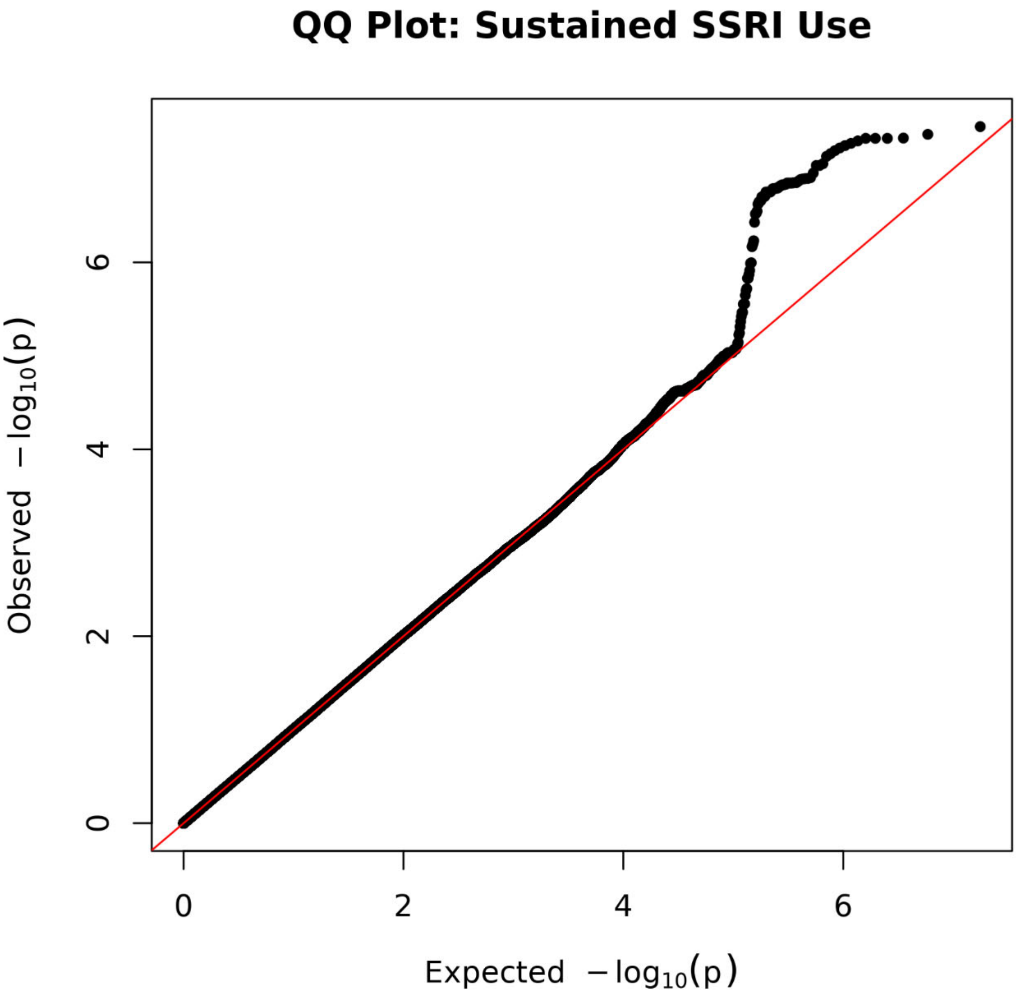

© 2026 Walker A et al. *JAMA Psychiatry*.

Supplementary Figure 18

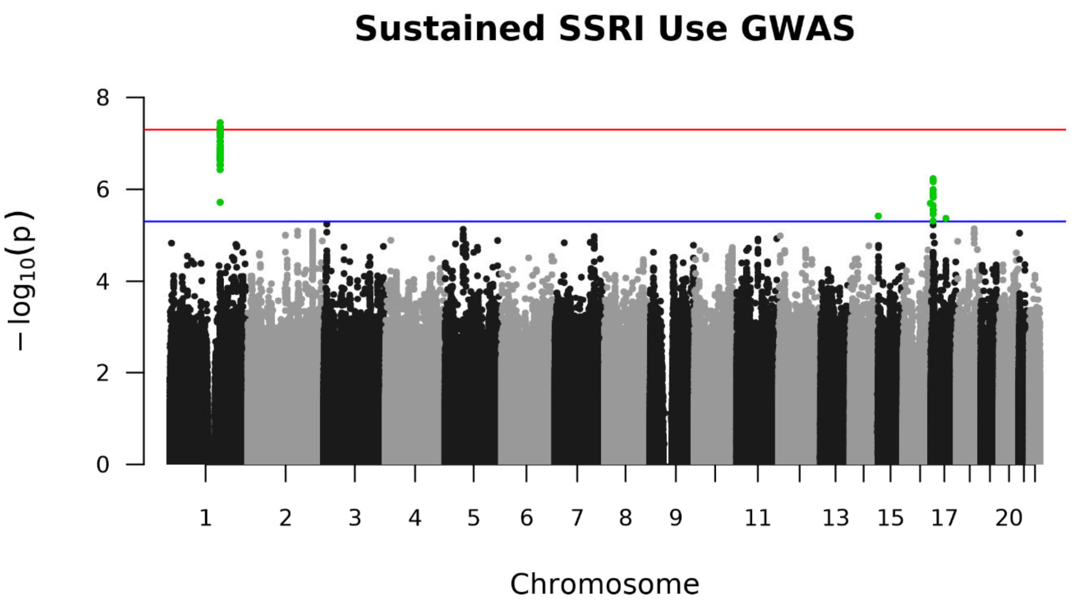

© 2026 Walker A et al. *JAMA Psychiatry*.

Supplementary Figure 19 and 20. Genome-wide association study (GWAS) of sustained SSRI/SNRI use. A logistic regression GWAS was conducted comparing participants with sustained SSRI/SNRI use (cases, n = 4,973) to those without (controls, n = 2,013). Controls were drawn from participants without sustained use (≥360 days) for any SSRI/SNRI and without self-reported bipolar disorder. Statistical analyses were performed using the same methods as the sustained SSRI use GWAS. Supplementary Figure 19: Quantile-quantile (QQ) plot and the genomic inflation factor ($\lambda = 1.001$) indicating minimal inflation. Supplementary Figure 20: Manhattan plot of all common SNPs. SNPs surpassing suggestive significance ($p < 5.0 \times 10^{-6}$) are highlighted in green. The SNP-based heritability was estimated at $h^2 = 0.13 \pm 0.077$ (mean ± SD), based on an observed phenotypic variance of 0.205 for the binary sustained SSRI use trait.

Supplementary Figure 19

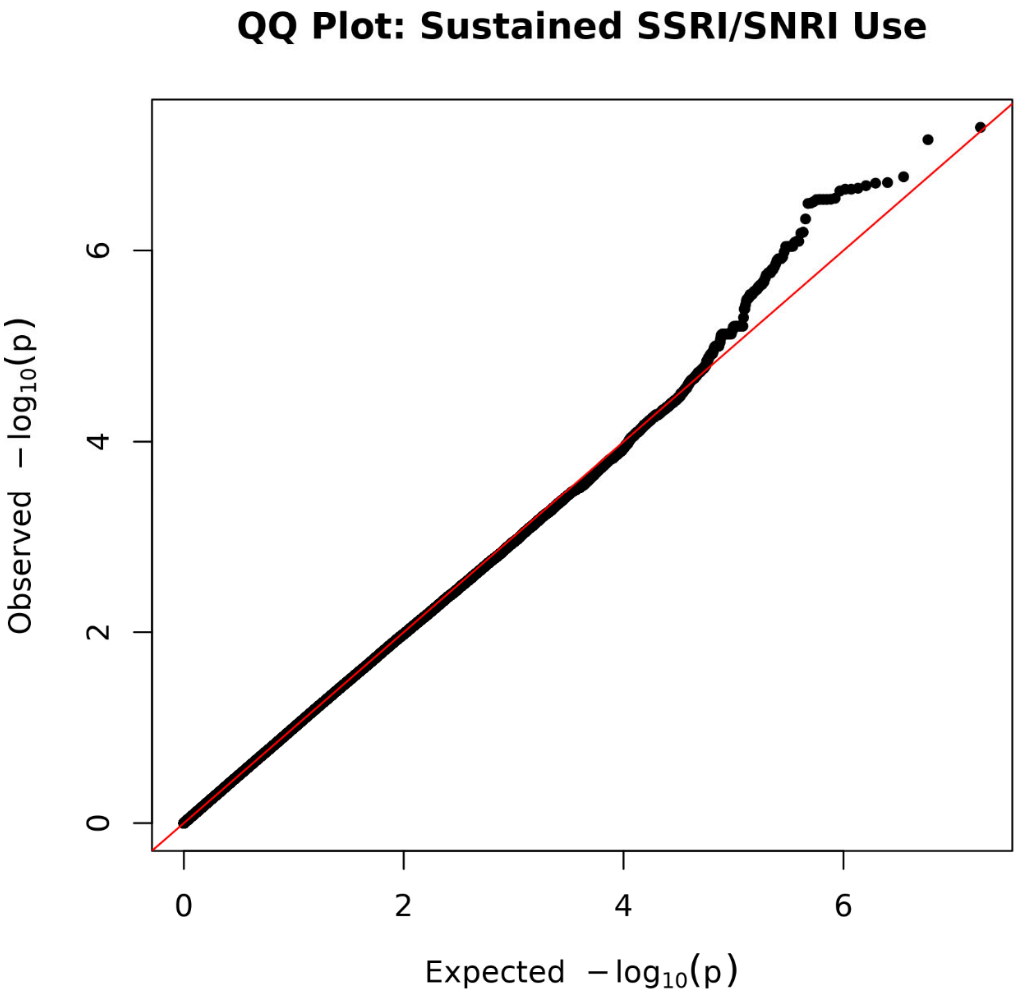

© 2026 Walker A et al. *JAMA Psychiatry*.

Supplementary Figure 20

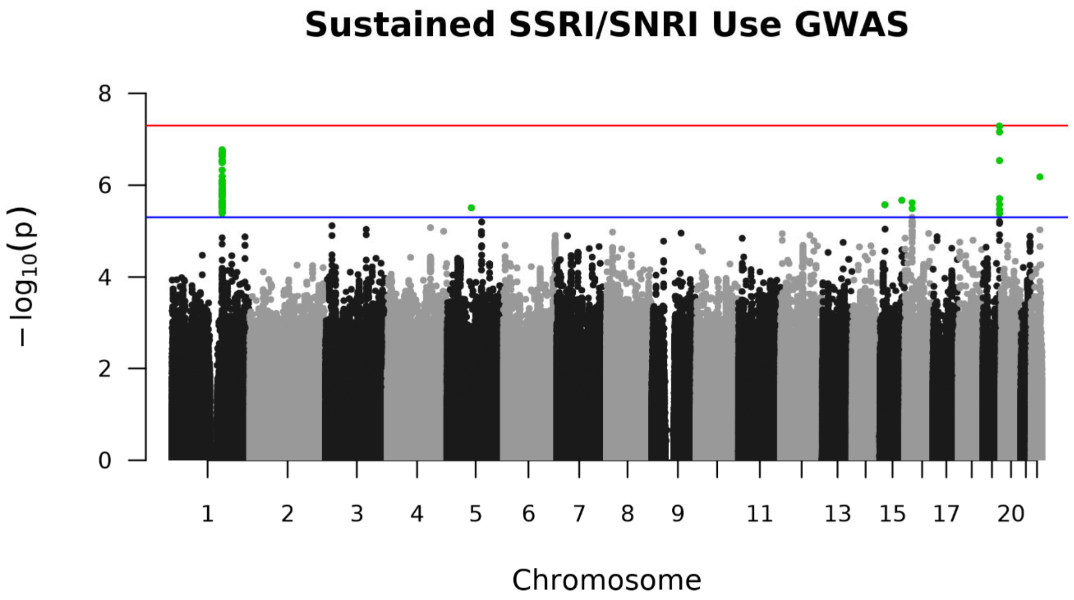

© 2026 Walker A et al. *JAMA Psychiatry*.

Supplementary Figures 21 and 22. Genome-wide association studies of self-reported antidepressant efficacy. Supplementary Figure 21: Results from a logistic regression GWAS comparing participants who reported efficacy with an SSRI (cases, *n* = 5,862) versus those who reported no efficacy (controls, *n* = 2,410). SNP-based heritability was estimated as $h^2 = 0.069 \pm 0.110$ (posterior mean ± SD), based on an observed phenotypic variance of 0.206 for the binary SSRI efficacy trait. The GWAS showed minimal genomic inflation (λ = 1.002), and the Manhattan plot highlights SNP-level associations across the genome. Supplementary Figure 22: Results from a logistic regression GWAS focused on SSRI/SNRI self-reported efficacy, comparing cases (*n* = 7,466) to non-efficacy controls (*n* = 806). SNP-based heritability was estimated as $h^2 = 0.004 \pm 0.011$ (posterior mean ± SD), based on an observed phenotypic variance of 0.088. There was no genomic inflation (λ = 0.999), and SNPs are visualized in a Manhattan plot.

Supplementary Figure 21

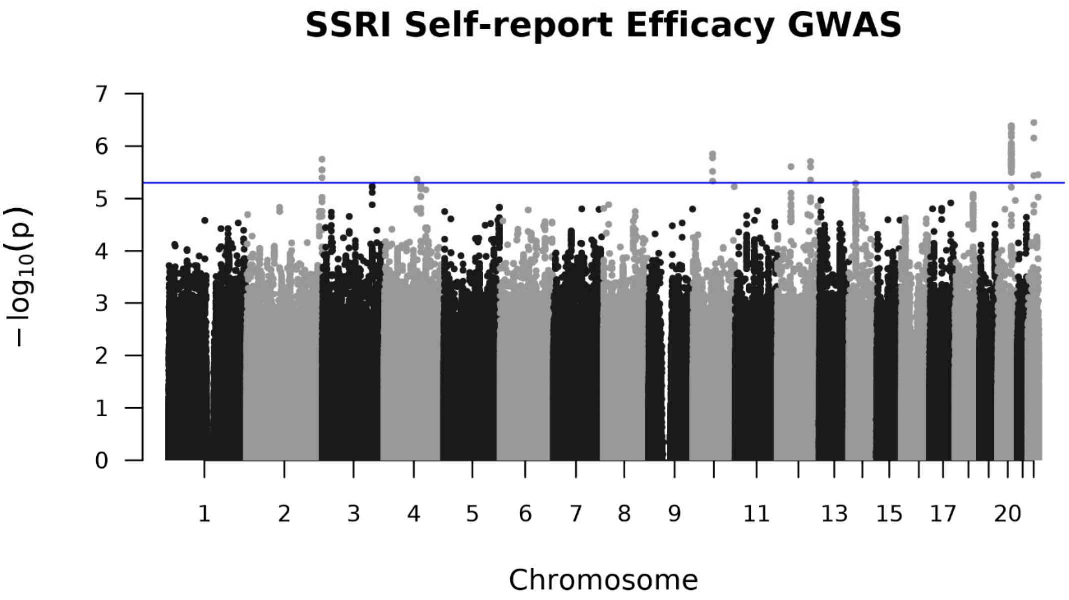

© 2026 Walker A et al. *JAMA Psychiatry*.

Supplementary Figure 22

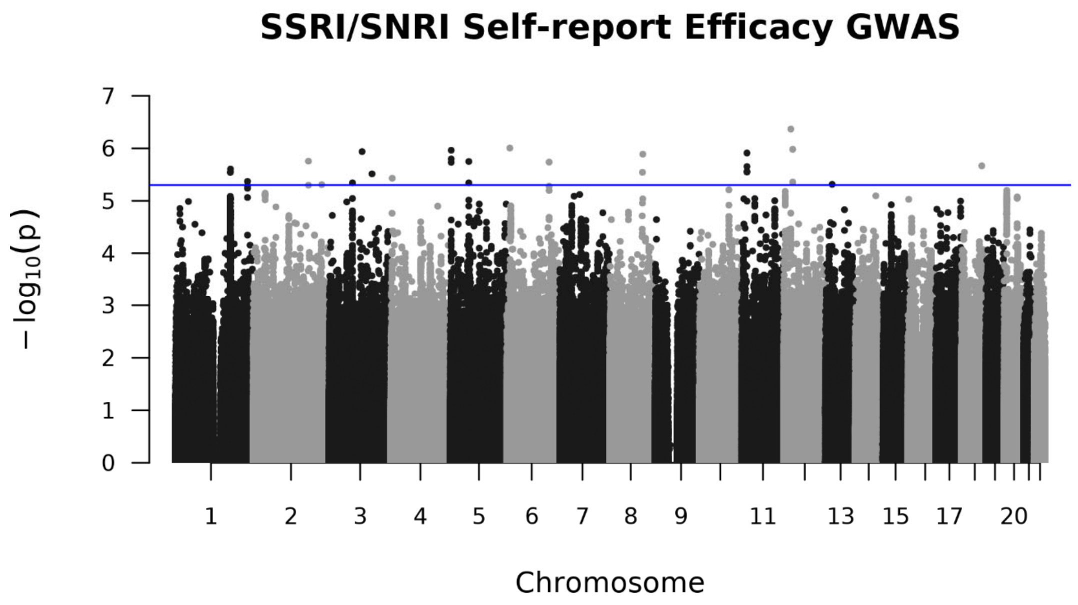

References

1. Crouse, J.J. *et al.* Evening chronotypes with depression report poorer outcomes of SSRIs: A survey-based study of self-ratings. *Biol Psychiatry* (2024).
2. Sanghani, H.R. *et al.* Patient fibroblast circadian rhythms predict lithium sensitivity in bipolar disorder. *Mol Psychiatry* **26**, 5252-5265 (2021).
3. McCarthy, M.J. *et al.* Chronotype and cellular circadian rhythms predict the clinical response to lithium maintenance treatment in patients with bipolar disorder. *Neuropsychopharmacology* **44**, 620-628 (2019).
4. Wu, Y. *et al.* GWAS of peptic ulcer disease implicates Helicobacter pylori infection, other gastrointestinal disorders and depression. *Nat Commun* **12**, 1146 (2021).
5. Zheng, Z. *et al.* Leveraging functional genomic annotations and genome coverage to improve polygenic prediction of complex traits within and between ancestries. *Nat Genet* **56**, 767-777 (2024).
6. Zeng, J. *et al.* Signatures of negative selection in the genetic architecture of human complex traits. *Nat Genet* **50**, 746-753 (2018).
7. Chang, C.C. *et al.* Second-generation PLINK: rising to the challenge of larger and richer datasets. *Gigascience* **4**, 7 (2015).
8. Tingley, D., Yamamoto, T., Hirose, K., Keele, L. & Imai, K. mediation: R Package for Causal Mediation Analysis. *Journal of Statistical Software* **59**, 1 - 38 (2014).
9. Gafoor, R., Booth, H.P. & Gulliford, M.C. Antidepressant utilisation and incidence of weight gain during 10 years' follow-up: population based cohort study. *BMJ* **361**, k1951 (2018).
10. Arterburn, D. *et al.* Long-Term Weight Change after Initiating Second-Generation Antidepressants. *J Clin Med* **5**(2016).

© 2026 Walker A et al. *JAMA Psychiatry*.

11. Fava, M., Judge, R., Hoog, S.L., Nilsson, M.E. & Koke, S.C. Fluoxetine versus sertraline and paroxetine in major depressive disorder: changes in weight with long-term treatment. *J Clin Psychiatry* **61**, 863-7 (2000).
12. Petimar, J. *et al.* Medication-Induced Weight Change Across Common Antidepressant Treatments : A Target Trial Emulation Study. *Ann Intern Med* **177**, 993-1003 (2024).
13. Langas, A.M., Malt, U.F. & Opjordsmoen, S. In-depth study of personality disorders in first-admission patients with substance use disorders. *BMC Psychiatry* **12**, 180 (2012).
14. Del Casale, A. *et al.* Psychopharmacological Treatment of Obsessive-Compulsive Disorder (OCD). *Curr Neuropharmacol* **17**, 710-736 (2019).
15. [NICE], N.I.f.H.a.C.E. Neuropathic pain - drug treatment: Prescribing Information: Duloxetine. (2024).
16. [NICE], N.I.f.H.a.C.E. Migraine - Mangagement - Sceniaro: Migraine in adults. (2024).
17. [NICE], N.I.f.H.a.C.E. Neuropathic pain - drug treatment - Prescribing Information: Amitriptyline. (2024).
18. Hirschfeld, R.M. The use of mirtazapine in difficult-to-treat patient populations. *Hum Psychopharmacol* **17 Suppl 1**, S33-6 (2002).
19. Xu, B. *et al.* Genetic Correlates of Treatment-Resistant Depression. *JAMA Psychiatry* (2025).
20. Riechelmann, R.P., Burman, D., Tannock, I.F., Rodin, G. & Zimmermann, C. Phase II trial of mirtazapine for cancer-related cachexia and anorexia. *Am J Hosp Palliat Care* **27**, 106-10 (2010).
21. Nutt, D.J. Tolerability and safety aspects of mirtazapine. *Hum Psychopharmacol* **17 Suppl 1**, S37-41 (2002).
22. Wichniak, A., Wierzbicka, A. & Jernajczyk, W. Sleep and antidepressant treatment. *Curr Pharm Des* **18**, 5802-17 (2012).
23. Gandotra, K., Chen, P., Jaskiw, G.E., Konicki, P.E. & Strohl, K.P. Effective Treatment of Insomnia With Mirtazapine Attenuates Concomitant Suicidal Ideation. *J Clin Sleep Med* **14**, 901-902 (2018).
24. Mulder, R.T., Joyce, P.R. & Luty, S.E. The relationship of personality disorders to treatment outcome in depressed outpatients. *J Clin Psychiatry* **64**, 259-64 (2003).
25. Cipriani, A. *et al.* Effectiveness of lithium in subjects with treatment-resistant depression and suicide risk: a protocol for a randomised, independent, pragmatic, multicentre, parallel-group, superiority clinical trial. *BMC Psychiatry* **13**, 212 (2013).
26. Scott, K., Khayachi, A., Alda, M. & Nunes, A. Prediction of Treatment Outcome in Bipolar Disorder: When Can We Expect Clinical Relevance? *Biol Psychiatry* (2025).
27. Visscher, P.M. *et al.* Statistical power to detect genetic (co)variance of complex traits using SNP data in unrelated samples. *PLoS Genet* **10**, e1004269 (2014).

© 2026 Walker A et al. *JAMA Psychiatry*.
